# A contractile injection system is required for developmentally regulated cell death in *Streptomyces coelicolor*

**DOI:** 10.1038/s41467-023-37087-7

**Published:** 2023-03-16

**Authors:** Maria Vladimirov, Ruo Xi Zhang, Stefanie Mak, Justin R. Nodwell, Alan R. Davidson

**Affiliations:** 1 Department of Biochemistry, University of Toronto, Toronto, Ontario Canada; 2 Department of Molecular Genetics, University of Toronto, Toronto, Ontario Canada

**Keywords:** Bacterial development, Bacteriophages, Cellular microbiology

## Abstract

Diverse bacterial species produce extracellular contractile injection systems (eCISs). Although closely related to contractile phage tails, eCISs can inject toxic proteins into eukaryotic cells. Thus, these systems are commonly viewed as cytotoxic defense mechanisms that are not central to other aspects of bacterial biology. Here, we provide evidence that eCISs appear to participate in the complex developmental process of the bacterium *Streptomyces coelicolor*. In particular, we show that *S. coelicolor* produces eCIS particles during its normal growth cycle, and that strains lacking functional eCIS particles exhibit pronounced alterations in their developmental program. Furthermore, eCIS-deficient mutants display reduced levels of cell death and altered morphology during growth in liquid media. Our results suggest that the main role of eCISs in *S. coelicolor* is to modulate the developmental switch that leads to aerial hyphae formation and sporulation, rather than to attack other species.

## Abstract (web summary)

Bacteria can use extracellular contractile injection systems (eCISs) to inject toxic proteins into eukaryotic cells. Here, Vladimirov et al. provide evidence that the main role of eCISs in *Streptomyces* is not to attack other species, but to modulate the complex *Streptomyces* developmental process.

## Introduction

**Fig. 1:**
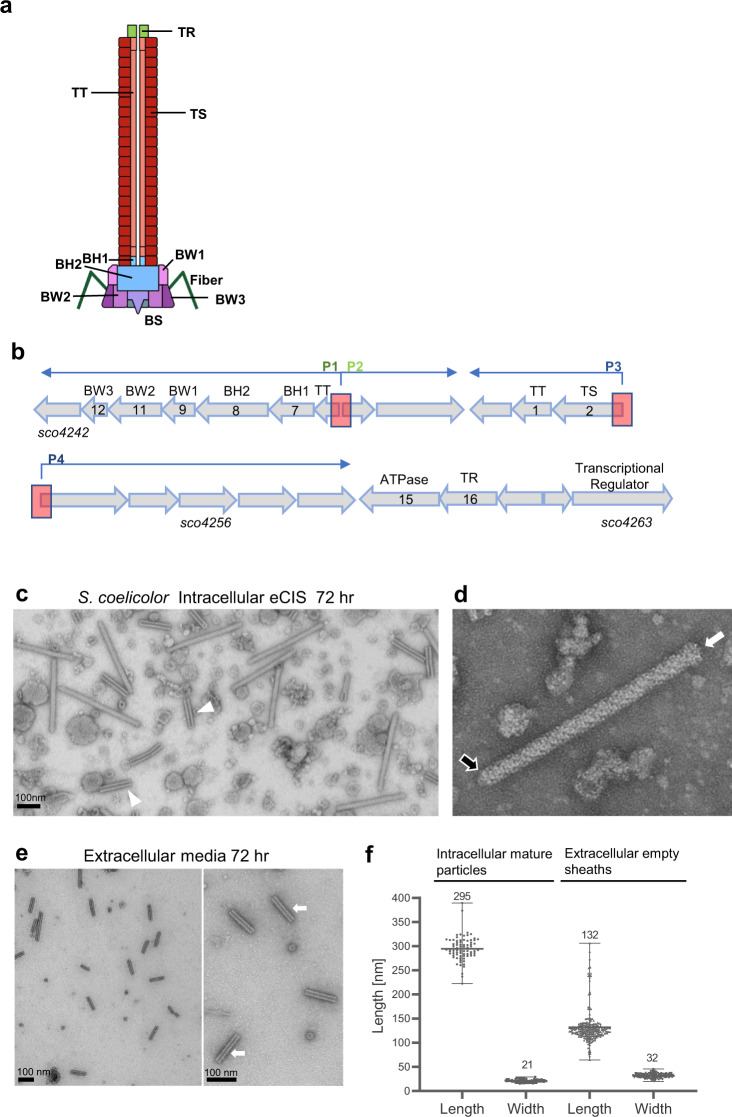
eCIS-derived particles can be visualized in cell lysates and extracellular media. **a** Schematic illustration of an eCIS particle. The diagram shows the conserved core structural components of an eCIS tail. Proteins are indicated by abbreviations (TR = tail terminator; TT = tail tube; TS = tail sheath; BH1, BH2 = baseplate hub components; BW1, BW2, BW3 = baseplate wedge components; BS = tail spike). **b**, Schematic representation of the eCIS cluster of *Streptomyces coelicolor* (*Sco*). Open reading frames and their transcriptional direction are represented by block arrows and are drawn to scale. Gene name is shown under the first and last genes in the cluster, spanning from *sco4242*-*sco4263*. The encoded eCIS function is shown as an abbreviation above each ORF. The Afp eCIS annotation, which is used in some other publications, is shown inside the arrows (Afp1-16^19^). The two divergent promoter regions are shown as red blocks. Forward and reverse transcriptional directions are shown in blue arrows above the cluster. **c**, eCIS were purified from lysates of *Sco* grown in liquid YEME media for 72 hr as described in Methods. Purified preparations were concentrated to 30X the concentration of the original culture. Transmission electron images were collected at 100,000X magnification. White arrowheads point to empty sheaths, present alongside with fully assembled eCIS. **d** A single assembled eCIS particle in its uncontracted extended conformation is shown. The white arrow points to the baseplate, and the black arrow shows the tail terminator complex. **e** Particles from the cell-free media of cultures grown as described in panel (**c**) were ultracentrifuged and concentrated to 30X the concentration of the original sample. Images collected at 80,000X magnification. White arrows point to typical emptied contracted tail sheath particles. Scale bar = 100 nm. **f** Scatter plot of the length and width of the eCIS-derived particles described in (**c**–**e**) (*n* = 80 for fully assembled uncontracted intracellular particles, *n* = 215 for extracellular contracted empty sheath particles). Within each plot, horizontal lines denote the mean values, which are also shown above the plot. The vertical borders denote the full range of value distribution.

Many strains of bacteria encode extracellular contractile injection systems (eCIS)^1–3^. eCIS are closely related to contractile-tailed bacteriophage (phage) tails. Like tails, they are composed of a long tube attached to a baseplate (Fig. 1a). The proteins comprising the eCIS tail-like structure are similar in sequence to phage proteins. However, genomic regions encoding eCIS are distinguished from phage tail regions by invariably encoding an AAA + ATPase of unknown function and a tail terminator protein (TR) that is a member of the *Pvc16_N* (PF14065) Pfam family^4,5^. They also often encode recognizable toxin proteins^2,6,7^. All of the eCIS with characterized functions mediate interactions between bacterial and eukaryotic cells. *Serratia* and *Photorhabdus* species release eCIS structures, known as Anti-feeding prophages (Afp) and Photorhabdus Virulence Cassettes (PVC), respectively, that mediate killing of insect cells^8,9^. This killing occurs through the injection of insecticidal toxins encoded downstream of the eCIS operon^8–10^. MACs (Metamorphosis Associated Contractile structures) are a distinct eCIS produced by the marine bacterium, *Pseudoalteromonas luteoviolacea*, that are required for the morphogenesis of the tubeworm *Hydroides elegans* in a mutualistic interaction^11^. The structures of several other eCIS have been examined in detail^12–14^, but their functions have not been determined.

eCIS regions have clearly been disseminated through horizontal gene transfer and this has resulted in their sporadic appearance across diverse bacterial clades^2,3^. A striking exception to this pattern is seen in the *Streptomyces* genus where the majority of species (>70%) encode at least one eCIS. *Streptomyces* propagate through a complex developmental program. Germinated spores initially grow to form extensive vegetative hyphae by a combination of tip extension and branching^15^. Upon nutrient deprivation or stress, programmed developmental and morphological changes result in the formation of aerial hyphae and subsequent formation of spores. This developmental switch is accompanied by a large shift in gene expression and the onset of secondary metabolite production, many of which are antibiotics^16,17^.

Given the uniquely complex lifestyle of *Streptomyces* and the prevalence of eCIS-encoding regions in this genus, we hypothesized that eCIS may play a fundamental role in the developmental program of these bacteria. To test this idea, we initiated studies on the eCIS-encoding region of the model strain *Streptomyces coelicolor* A3(2) (*Sco*), which has a single eCIS-encoding region. The goals of these experiments were to determine whether eCIS are produced as part of the normal *Sco* lifecycle and what role these little characterized entities might be playing.

## Results

### eCIS tail-like structures can be purified from *Sco* lysates

eCIS particles are built from a conserved set of structural proteins that are homologous to proteins found in contractile-tailed phages (Fig. 1a)^18^. The tubular portion comprises a tail tube (TT) coated by a sheath (TS) that mediates contraction. The tube is attached on one end to a baseplate constructed by attaching six wedges (composed of BW1, BW2 and BW3 proteins) to a hub containing the BH1 and BH2 proteins. The other end of the tube is capped by the terminator protein (TR). Cell surface attachment is usually mediated by fibers attached to the baseplate. Genes encoding all of the conserved eCIS structural proteins and an AAA + ATPase, which is also conserved in eCIS-encoding regions, are found in one region of the *Sco* genome (Fig. 1b).

To determine whether wild-type (WT) *Sco* produces eCIS particles, cell lysates of *Sco* cultures grown in liquid medium for 72 hr were subjected to a standard eCIS purification protocol (see Methods). Examination of the resulting purified samples using transmission electron microscopy (TEM) revealed abundant phage tail-like particles with average lengths of 295 nm and widths of 21 nm (Fig. 1c, d, f). These particles closely resemble previously observed eCIS particles with a rounded cap on one end and a baseplate on the other as indicated by arrows (Fig. 1d)^5,19,20^. The baseplate is somewhat smaller than other eCIS and fibers, which are attached to the baseplates of some eCIS^5,11,21^, were not detectable. The purified samples also contained many empty sheath structures of varying length (Fig. 1c, white arrowheads).

Strikingly, TEM micrographs of the extracellular fraction (supernatant collected after cell pelleting) revealed only what appeared to be emptied-out sheaths left behind after eCIS contraction (Fig. 1e). These structures were 100–150 nm long and 28–35 nm wide with a typical striation pattern characteristic of polymerized tail sheath, but they had no density in the center and were wider than mature wild-type eCIS tails (Fig. 1f). Tail sheath (TS) protein was also detected in the extracellular (E) fraction by western blot (Supplementary Fig. 1a, b, d). The strictly cytoplasmic protein, ActR, was not detected in the extracellular fraction (Supplementary Fig. 1b, d), suggesting that the appearance of TS protein in this fraction was not due to widespread cell lysis. *Sco* strains bearing deletions in the genes encoding either the tail sheath (ΔTS) or the baseplate wedge proteins (ΔBP) displayed no tail-like structures in extracellular samples or cellular lysates. Western blots confirmed the absence of these proteins from the mutant strain preparations (Supplementary Fig. 1a, c, d).

To investigate the role of the ATPase in the formation of eCIS particles, we generated a deletion in the *Sco* AAA + ATPase gene (*sco4259*) and performed TEM experiments as described above on lysates of this mutant. The eCIS particles produced appeared identical to those produced by the WT strain (Supplementary Fig. 2a). Similar levels of extracellular empty sheaths were also observed (Supplementary Fig. 2b, c). These results imply that the ATPase is not required for the production of eCIS-like particles with normal appearance and ability to contract. This finding agrees with work on the *Photorhabdus* eCIS^5^, which also showed that normal-looking eCIS particles are formed in the absence of the ATPase^5^.

We used mass spectrometry to determine the composition of the purified tail-like particles produced by *Sco*. As expected, most of the eCIS structural proteins (Fig. 1a) that have phage tail homologues were detected in all three purifications (TR, TS, TT, BH2, BW2) while others of these conserved structural proteins (BH1 and BW1) were detected in at least one of three separate purifications performed (Supplementary Tables 1 and 2). BW3 was the only conserved structural protein that was not detected in any purifications. Two proteins of unknown function, encoded by genes *sco4242* and *sco4256*, were detected in all three purifications, and one other, encoded by *sco4251*, was detected in at least one purification. The AAA + ATPase was not detected in purified eCIS particles. We also performed mass spectrometry on eCIS-related particles purified from the extracellular fraction. In this case we detected only TS and TT proteins, which are the likely components of the empty contracted sheaths observed by TEM. Importantly, purified preparations produced from the ∆BP mutant contained only TT and TS proteins, demonstrating that the detection of most eCIS proteins requires the proper eCIS assembly, which cannot occur in the absence of the baseplate wedge proteins^5^. Aggregates of TS and TT protein may still be purified by high-speed centrifugation. Overall, these mass spectrometry experiments show that the tail-like particles that we have purified are indeed produced from the eCIS region of *Sco* and that the composition of these particles is the same as other eCIS.

### eCIS do not contribute to killing of other species by *Sco*

**Fig. 2:**
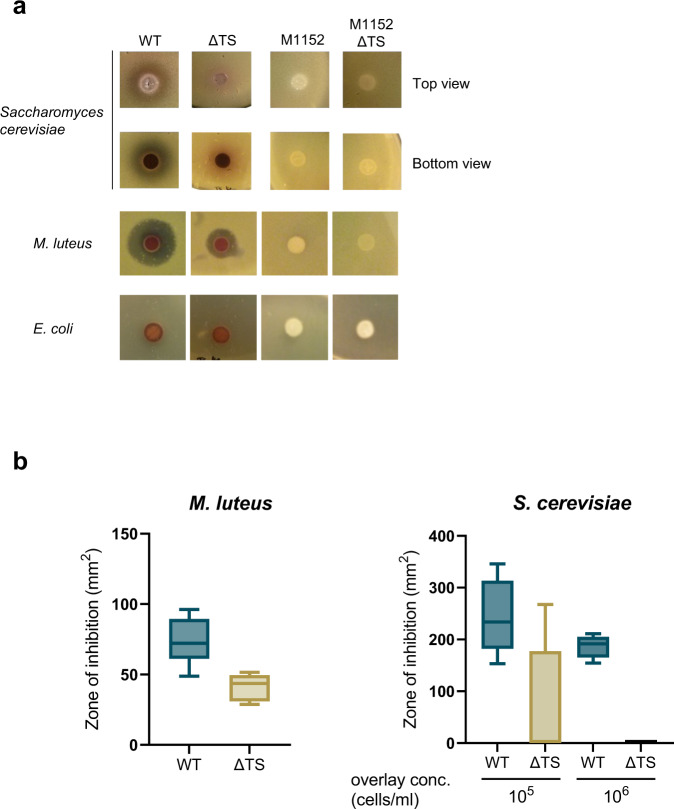
Interspecies growth inhibition induced by WT and eCIS-deficient *Sco* strains. Assays were performed by spotting on agar plates 10^5^ SFU of *Sco* wild-type (WT), tail sheath knockout (ΔTS), M1152 (a mutant that does not produce the four major *Sco* antibiotics), or M1152 harboring a tail sheath knockout mutation (M1152 ΔTS). Cells were grown for 3 days before being overlaid with species to be tested as indicated in each panel. **a** Zones of clearing around the *Sco* colonies indicate lethality or growth inhibitory activity against the indicator lawn. All of the results shown for a tested strain were obtained from the same agar plate. **b** The inhibitory activity of *Sco* M145 strains was quantified by calculating the surface area of the zone of clearance around each colony. Results are shown as box plots. The center horizontal line denotes the median and boxes extend from the 25^th^ to the 75^th^ percentile of values, whiskers show the full range of value distribution per strain. *n* = 6 biologically independent experiments in assays against *M. luteus*, *n* = 8 in assays against *S. cerevisiae* (10^5^) and *n* = 6 in assays against *S. cerevisiae* (10^6^). M1152 strains did not show any inhibitory activity against *M. luteus* or *S. cerevisiae*. Source data are provided in the Source Data file.

A previous study implicated the eCIS of *Streptomyces lividans* as being responsible for a growth inhibitory activity against the yeast, *Saccharomyces cerevisiae*^22^. Since the components of *S. lividans* eCIS are very similar in sequence to those of the *Sco* system, it was of interest to determine whether the *Sco* eCIS also displayed this activity. As shown in Fig. 2a, we found that WT *Sco* did indeed inhibit the growth of *S. cerevisiae* and that this activity was not seen for the ∆TS strain. As *Sco* is known to produce several antibiotics that might contribute to the observed growth inhibition, we tested the ability of *Sco* strain M1152^23^, in which genomic regions required for synthesis of the four major *Sco* antibiotics are deleted (Δ*act*, Δ*red*, Δ*cda*, Δ*cpk*), to inhibit yeast growth. We confirmed that this strain still produces eCIS-related particles (Supplementary Fig. 3a). We found that neither strain M1152 nor a mutant version of this strain that does not produce eCIS (M1152∆TS) inhibited yeast growth (Fig. 2a). These results imply that antibiotics are required for the growth inhibition of *S. cerevisiae* and that the absence of eCIS may indirectly affect the inhibitory activity by perturbing antibiotic production.

Similar to our results with *S. cerevisiae*, we found that *Sco* inhibited growth of the Gram-positive bacterial species *Micrococcus luteus*, and that this inhibition was partially attenuated in the ∆TS strain (Fig. 2a, b). However, strain M1152 and the ∆TS mutant lacked this activity. Finally, we found that *Sco* inhibited the growth of four different *Streptomyces* species, but the ∆TS strain displayed the same level of inhibition, implying that the eCIS was not required for the growth inhibition (Supplementary Fig. 3b). In summary, these growth inhibition experiments demonstrate that *Sco* does inhibit the growth of some bacterial species and *S. cerevisiae*, but there does not appear to be a direct role for the eCIS in this activity.

### eCIS mutants exhibit decreased hyphal death and reduced pellet size during differentiation in liquid cultures

**Fig. 3:**
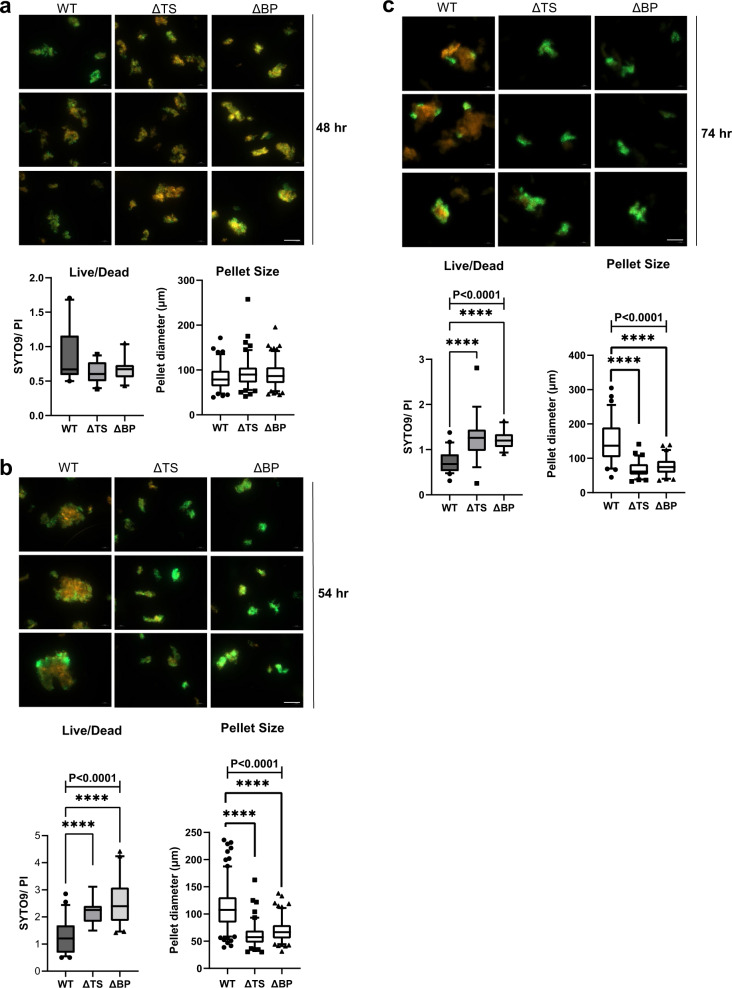
*Sco* eCIS mutants exhibit reduced cell death during hyphal differentiation. *Sco* wild-type (WT), tail sheath knockout (ΔTS) or baseplate knockout (ΔBP) strains were inoculated into 50 ml of YEME media at a final concentration of 10^7^ SFU /mL. At **a**, 48 hr, **b**, 54 hr, or **c**, 74 hr post-inoculation, samples were stained with equal proportions of SYTO 9, a membrane permeant nucleic acid stain, and propidium iodide, which stains only dead or damaged cells. Therefore, dead and live cells are visualized by red and green fluorescence, respectively. The ratio of live/dead cells was calculated from total fluorescence intensity values per image. Diameters of individual mycelial pellets were manually measured (for WT, ΔTS and ΔBP, respectively; at 48 h – *n* = 99, 116, 131, at 54 h – *n* = 173, 113, 138 and at 74 h – *n* = 73, 69, 71). Results are shown as box plots. The center horizontal line denotes the median and boxes extend from the 25^th^ to the 75^th^ percentile of values. Whiskers denote values within the 5–95^th^ percentile range, with individual data points shown for the values outside that range. One-way ANOVA and Dunnett’s multiple comparisons test were performed to calculate statistical significance. Adjusted *P*-values are shown above the relevant graphs. In (**b**), *P* value for live/dead ratios = 2.96E-06 (WT vs. ΔTS), 1.33E-13 (WT vs. ΔBP), *P* value for pellet size = 2.9E-31 (WT vs. ΔTS), 1.1E-26 (WT vs. ΔBP). In (**c**), *P* value for live/dead ratios = 8.2E-05 (WT vs. ΔTS), 9.5E-08 (WT vs. ΔBP), *P* value for pellet size = 4.9E-20 (WT vs. ΔTS), 4.2E-17 (WT vs. ΔBP). Images shown for each panel are representative of at least three independent experiments. Scale bar = 100 µm. Source data are provided in the Source Data file.

Given the apparent lack of killing of other species by the *Sco* eCIS, we postulated that eCIS might mediate an intra-strain cell-killing activity during the normal developmental program. To visualize and quantify levels of cell death during *Sco* differentiation, we used a well-established bacterial viability assay^24^. Samples are stained with SYTO 9, which labels both live and dead cells, and with propidium iodide (PI), which penetrates and stains only bacteria with damaged membranes, which are likely dead. In the presence of both stains, live bacteria appear green, whereas dead bacteria appear red. After 48 hr of growth in liquid media, WT *Sco* grew mycelial pellets as has been previously observed^25,26^. The average diameter of these pellets was 85 µm (Fig. 3a). The same size pellets were seen in the eCIS-deficient strains. By quantitating the staining by SYTO9 and PI, we calculated the ratio of live to dead cells in the WT cultures to be 0.8 (Fig. 3a). The live/dead ratios were slightly less in the mutant cultures though the difference was not significant. Differences between the WT and the eCIS-deficient strains became evident at the 54 and 74 hr of growth points. WT samples developed many large mycelial pellets ranging from 100 to 300 µm in diameter that displayed increased PI staining in the center (Fig. 3b, c). By contrast, in both eCIS-deficient mutants, the preponderance of observed hyphal pellets remained small with the average pellet sizes being half that of WT *Sco*. In addition, the live/dead cell ratios of the mutants were double those of WT (Fig. 3b, c). Overall, these data show that the development of WT *Sco* in liquid media is accompanied by formation of large mycelial pellets and considerable levels of cell death. Mutants lacking eCIS particles significantly deviate from this behavior displaying smaller cell pellets and a much lower frequency of cell death.

To address the role of the AAA + ATPase protein in eCIS function, we performed the same experiment as described above using the ∆ATPase *Sco* mutant strain. Although this strain produces eCIS particles with a normal appearance, the mycelial pellets produced by this strain after 54 h of growth in liquid media resembled those produced by the eCIS-deficient mutant strains. These pellets were approximately half the size of the WT pellets, and they displayed half the frequency of cell death (Supplementary Fig. 4b). This trend continued at the 74 h time point (Supplementary Fig. 4c). As with the other eCIS-deficient mutants, the mycelial pellets of the ∆ATPase strain resembled WT after 48 h of growth (Supplementary Fig. 4a). These data demonstrate that the presence of tail-like particles alone does not mediate cell death and that the ATPase performs a function that is required for the biological activity of the eCIS particles.

### *Sco* strains lacking eCIS display altered development

**Fig. 4:**
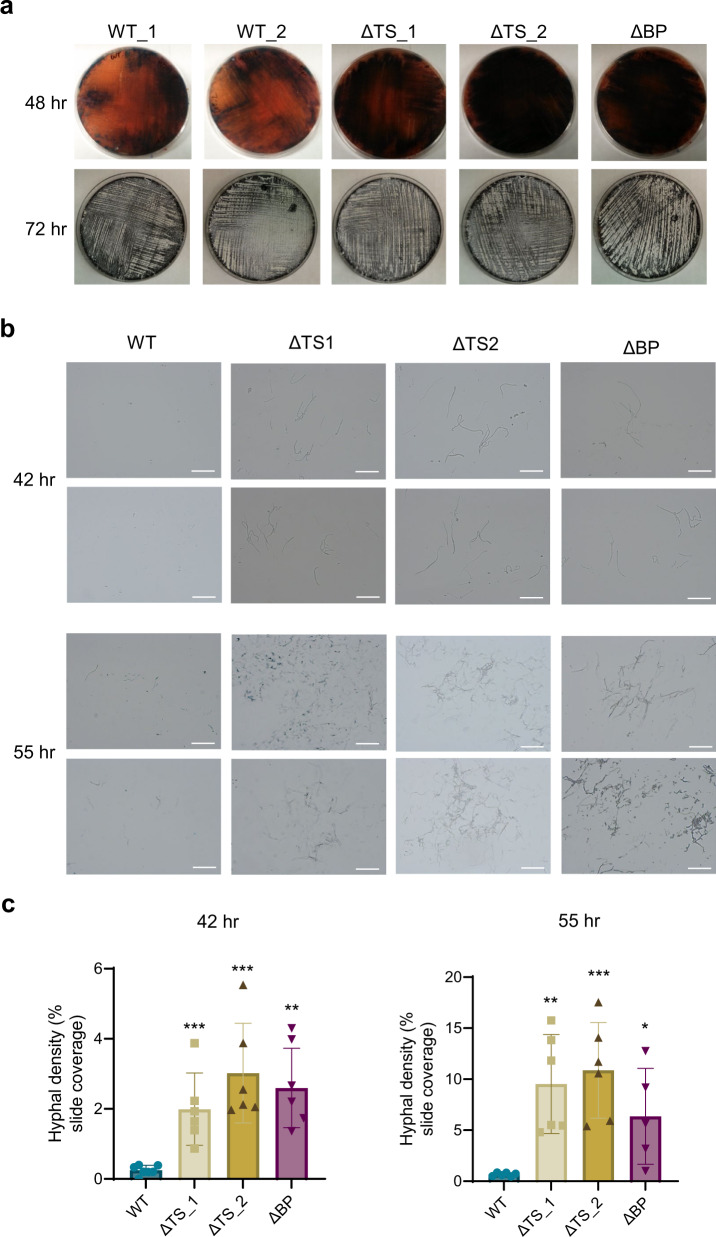
eCIS mutants display accelerated development and early spore formation on solid medium. **a** 10^8^ SFU of *Sco* wild-type (WT), two independently isolated tail sheath knockout strains (ΔTS_1 and ΔTS_2) or a baseplate knockout strain (ΔBP) were plated on R2YE agar. Top panel shows representative images of secondary metabolite development in lawns grown for 48 h; bottom panel shows aerial hyphae development on the same plates as above, at 72 h. **b** Representative transmission light micrographs of surface imprints on cover slips of agar-grown WT *Sco*, ΔTS or ΔBP mutant strain lawns 42 and 55 h postgermination. The aerial hyphae and spores stick to the coverslip, but not the vegetative hyphae. Two fields are shown for each sample. Results are representative of three independent experiments with two biological replicates per strain. Scale bar = 20 µm. **c** The aerial hyphae density of the surface imprints on coverslips described in (b) was quantified through image analysis of light micrographs (*n* = 6 samples measured for each strain, at each time point). Graphs show the mean value with error bars representing standard deviation of the mean. Mean density values of eCIS mutants were compared to WT using a two-sample t-Test, two-sided *P*-values are shown; * *P* < 0.05, ** *P* < 0.01, *** *P* < 0.001. *P* values = 0.0009, 0.0003 and 0.0024 for ΔTS_1, ΔTS_2 and ΔBP 42 h hyphae samples, respectively. *P* values = 0.0012, 0.0003 and 0.0148 for ΔTS_1, ΔTS_2 and ΔBP 55 h hyphae samples, respectively.

*Sco* follows a developmental program characterized by a shift from formation of vegetative hyphae to aerial hyphae, which is followed by spore formation. To determine whether the lack of eCIS might perturb this program, we compared the growth of the WT and ∆TS strains on solid media. At early stages of growth, visual examination of the agar plates did not reveal any significant differences, with all strains producing similar vegetative lawns. However, after 48 h, eCIS mutants had visibly developed faster in terms of producing the pigmented secondary metabolite, actinorhodin (Fig. 4a, top panel). At later time points, any visible apparent differences disappeared, and by 72 h all strains had developed comparable lawns of aerial mycelia (Fig. 4a, bottom panel). In liquid media, we observed that WT and eCIS-deficient strains exhibited similar growth rates (Supplementary Fig. 5).

It should be noted that the production of actinorhodin by eCIS-deficient strains was variable depending on the media used. In some conditions of liquid or solid media growth, these mutants produced considerably less pigment than WT (Supplementary Fig. 6a–c). Since the regulation of actinorhodin production is complicated^27–31^, it is difficult to rationalize these variable effects. However, the perturbation of secondary metabolite production in the absence of eCIS is consistent with a role for it in the developmental program.

We used a microscopy approach to gain a clearer assessment of aerial hyphae formation at intermediate time points in the developmental program. We applied glass coverslips to the surface of the growing bacterial lawn. Since the surface of the coverslips is hydrophobic, only aerial hyphae, which have a hydrophobic coating, stick to them. Adhered hyphae were then detected through microscopic examination. No cells were seen on coverslips that had been applied to the surface of lawns in any of the samples up to 35 hr post-germination. At 42 hr, the WT strains had yet to produce any visible aerial hyphae, while the ∆TS and ∆BP mutant strains showed well-developed aerial hyphal strands clustered in numerous regions on the microscope slide (Fig. 4b, c). This trend continued at 55 hr of growth where the WT strains had developed a few dispersed hyphal strands compared to the eCIS-deficient mutants, which displayed larger, more developed clusters of hyphae (Fig. 4b, c).

### eCIS-encoding genes are developmentally regulated

**Fig. 5:**
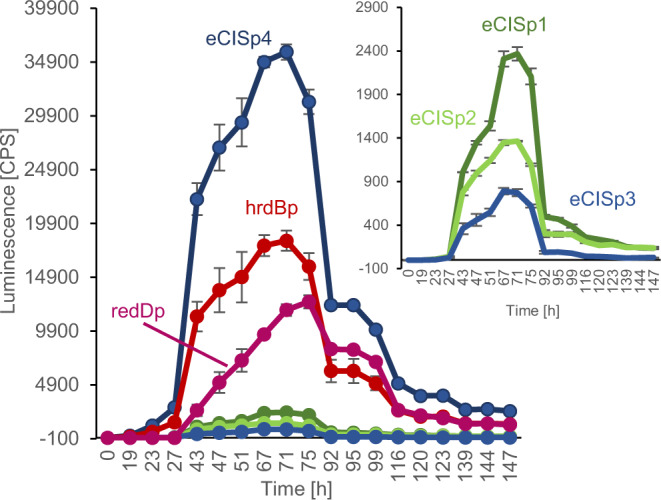
The eCIS cluster of *S. coelicolor* is expressed during vegetative growth right before the developmental switch. A fusion construct of each eCIS promoter (eCISp1-p4, corresponding to promoters P1-P4 shown in Fig. 1b) with *luxCDABE* was conjugated with *Sco* and grown on 96-well MS agar plates for 7 days. *hrdBp* and *redDp* reporter strains were used as controls. Luminescence was measured three times a day. Activity of the weaker promoters: eCISp1, eCISp2 and eCISp3 is shown in enlarged view in the top right insert. Three biological replicates were tested for each assay (*n* = 3). The mean normalized to empty vector is shown with error bars representing standard deviation of the mean. Source data are provided in the Source Data file.

Since the absence of eCIS appeared to perturb the *Sco* developmental program, we wondered if the eCIS-encoding genes were expressed in a developmentally regulated pattern. To this end, we fused four known eCIS region promoters (Fig. 1b; Supplementary Fig. 7), to plasmid-borne genes encoding luciferase activity (*luxCDABE)*^32^. Introduction of these plasmids into WT *Sco* revealed that all four eCIS promoters were active and displayed the same temporal pattern of expression (Fig. 5). This pattern was similar to the promoter of the major sigma factor gene *hrdB*^33^, which acts during vegetative growth and to the promoter of *redD*. This gene encodes the transcriptional regulator of the *red* biosynthetic cluster, which is transcribed at the early stages of the developmental switch^34,35^. These results show that the eCIS operons are expressed during the vegetative growth phase under normal growth conditions, right before the developmental switch that leads to aerial hyphae formation and sporulation.

Our observed transcriptional pattern for the eCIS promoters is consistent with previous observations that eCIS expression is regulated by *bldA*^36–38^. This gene is required for proper morphological development, secondary metabolism, and sporulation^39–41^. It encodes the only tRNA for the rare leucine codon, TTA, present in 2-3% of *Streptomyces* genes. The production of the putative eCIS transcriptional regulator encoded by *sco4263* is dependent on BldA as it contains a TTA codon. We analyzed 56 *Streptomyces* eCIS regions and found that 42 contained at least one gene containing an in-frame TTA codon. Of the identified TTA-containing genes, 22 are putative eCIS regulatory genes (Supplementary Data 1 and 2). These findings indicate that eCIS regions are often regulated by *bldA*, which ensures that eCIS particles will accumulate in conjunction with the morphogenetic transition.

### Identification of a putative toxin and fiber for *Streptomyces* eCIS

To address the question of whether eCIS may perform a shared function among many *Streptomyces* species, we sought to identify conserved features particular to *Streptomyces* eCIS. To this end, we searched publicly available *Streptomyces* genomes and identified 153 eCIS-encoding regions within 127 *Streptomyces* genomes (Supplementary Data 3). eCIS have been classified previously into 6 distinct subtypes based on sequence similarity and gene arrangement^3^. The majority (>80%) of *Streptomyces* species including *Sco* encode a type IId eCIS, as has been previously noted^3^; thus, we assume that any conserved eCIS function must be performed by this type.

**Fig. 6:**
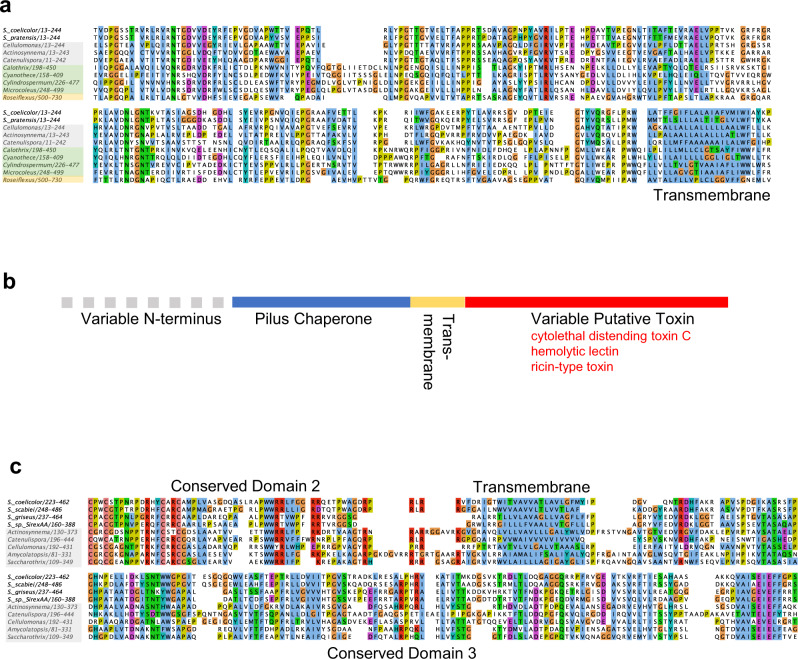
Conservation of the putative toxin and fiber of the *Sco* eCIS. **a** A diverse selection of hits from a BLAST search initiated with Sco4256, the putative toxin, were aligned and analyzed by HHpred. Sequences shown are from *Streptomyces* species (unshaded), other Actinomycete species (shaded gray), Cyanobacteria (shaded green), and Chorflexi (shaded yellow). All proteins are associated with type IId systems and all genera are filamentous except for *Cyanothece*. **b** A schematic of the conserved domains of proteins related to Sco4256 is shown. **c** A diverse selection of hits from a BLAST search initiated with Sco4242, the putative fiber, were aligned. Sequences shown are from *Streptomyces* species (unshaded), other Actinomycete species (shaded gray). These proteins are found in type IId eCIS. All alignments were performed with MUSCLE^62^ as implemented in Jalview^63^. The clustal coloring scheme was used.

Most eCIS regions encode proteins with domains associated with toxin activity. These proteins are thought to be assembled into the eCIS particle and injected into target cells to mediate cell killing^10,42,43^. We were not able to identify a protein encoded frequently within *Streptomyces* eCIS regions that contained an identifiable toxin domain. However, a BLAST^44^ search with the Sco4256 protein, which we found to be a component of the *Sco* eCIS particle (Supplementary Table 1), revealed closely related proteins encoded in 90% of the 105 *Streptomyces* class IId eCIS (Supplementary Data 4). Alignment of a representative group of sequences and structural analysis using HHpred^45^ suggested a three domain architecture with the second domain predicted to be a transmembrane helical region (Fig. 6a; Supplementary Data 5). The first domain, present across all homologues, shows high probability similarities to periplasmic chaperones involved in pilus assembly among other structures and families (Supplementary Data 5). Approximately one third of homologues, including the one from *Sco*, possess a C-terminal third domain with similarity to various bacterial toxins and hemolytic lectins (Fig. 6b; Supplementary Data 5). These proteins may be delivered by eCIS as toxic cargo. Interestingly, homologues of these proteins were also identified in class IId eCIS regions in genomes of other Actinobacterial species and several species of filamentous Cyanobacteria and Chloroflexi (Fig. 6a; Supplementary Data 4). Proteins with highly significant similarity to Sco4256 were rarely found in non-eCIS encoding regions.

Structurally characterized eCIS^5,13,20^ and contractile phage tails^1^ have fiber proteins attached to their baseplates. In phages, these fibers mediate host cell attachment. In most contractile-tailed phage genomes and in the genomes of characterized eCIS^8,11,13,14^, fiber proteins are encoded immediately downstream of genes encoding the baseplate wedge proteins, BW2 and BW3. In the *Sco* eCIS region, the protein product of the gene in this position (*sco4242*) (Fig. 1b) was found to be a component of the eCIS particle (Supplementary Table 1). A BLAST search with this protein revealed closely related homologues in 75% of class IId eCIS regions in *Streptomyces*, and in the class IId eCIS of other Actinomycete species (Fig. 6c; Supplementary Data 6). Alignment of these proteins revealed a conserved C-terminal region approximately 150 residues long, which is preceded by a strongly predicted transmembrane motif (Fig. 6c). The C-terminal domain has strong similarity to carbohydrate-degrading domains, as predicted by HHpred (Supplementary Data 7). Since receptor-binding proteins of phages, of which fibers are one group, often bind to or degrade carbohydrates on the bacterial cell surface, these predicted functions of the Sco4242 protein are consistent with its serving as a bacterial cell surface binding protein. Conserved domain 2, comprising approximately 50 residues immediately preceding the transmembrane segment, is Cys-rich and may be a Zn-binding domain as predicted by HHpred (Supplementary Data 7). This region might mediate the attachment of these proteins to the baseplate. In summary, our bioinformatic data (summarized in Supplementary Data 1) show that most *Streptomyces* type IId eCIS share related putative fiber and toxin proteins, supporting a common function for eCIS in this genus.

## Discussion

eCIS particles are produced by bacterial species in widely divergent bacterial clades. The dispersed occurrence of eCIS and their demonstrated roles in mediating toxic activities against eukaryotic cells has led to a view of these systems as defense mechanisms, useful under specific conditions but not central to normal cellular function. The results presented here introduce an entirely distinct role for eCIS in *Sco* where they appear to participate in the developmental process of this species. In particular, we have shown that *Sco* produces eCIS particles as part of its normal growth cycle and that strains lacking functional eCIS particles exhibit pronounced alterations in their developmental program. These changes included perturbation of antibiotic production during growth on solid and in liquid media (Fig. 4a; Supplementary Fig. 6), and accelerated development of aerial hyphae on solid media (Fig. 4b, c). Most intriguingly, eCIS-deficient mutants displayed significantly reduced levels of cell death and altered morphology during liquid growth (Fig. 3). These results suggest that *Sco* eCIS function by inducing intra-strain lethality, which may play a role in the developmental process.

In contrast to a previous study on *Streptomyces lividans*, we did not detect eCIS-mediated inhibition of *S. cerevisiae* growth by *Sco*^22^. Given that the eCISs in these two species are very closely related, we would expect them to perform the same function. It is possible that we did not detect the growth inhibitory activity of the *Sco* eCIS because we used a different assay that may not be as sensitive as that used in the other study. In addition, since we observed complicated changes in antibiotic production by the eCIS-deficient strains, it is possible that indirect effects on antibiotic production caused the effects seen in the previous study. This idea is supported by the absence of *S. cerevisiae* growth inhibition induced by strain M1152 (Fig. 2a), which does not produce the four major *Sco* antibiotics. Casu et al.^46^ also recently found that the *Sco* eCIS played no role in inhibiting the growth of other bacterial species or *S. cerevisiae*.

While intracellular *Sco* lysates displayed mostly fully sheathed eCIS structures with typical appearance (Fig. 1c), extracellular samples almost exclusively contained empty contracted sheath species (Fig. 1e). This stark difference between the intracellular and extracellular pools of eCIS-derived structures suggested that eCIS were not escaping from cells through generalized lysis, a conclusion also supported by the absence of ActR, a cytoplasmic protein, in the extracellular fractions containing sheath proteins (Supplementary Fig. 1b, d). A model that could account for the above observation is that in intact mycelia the tail-like structures are membrane-bound. Upon encountering the appropriate stimulus, the eCIS may extend through the membrane to contact a neighboring cell, resulting in contraction and release of the contracted sheath. The *Sco* eCIS at 300 nm in length are considerably longer than other eCIS, which are approximately 120 nm^5,14,20^ and contractile-tailed phages infecting Gram-positive bacteria possess tails of up to 200 nm in length^47^. Thus, a membrane-bound *Sco* eCIS is theoretically long enough to extend through the membrane and cell wall of its host cell to contact a nearby cell. The possibility of the *Sco* eCIS being attached to the cell membrane is also supported by the conserved presence of a strongly predicted transmembrane helix in its putative fiber protein (Fig. 6c). In a recent study on *Anaboena*, a filamentous cyanobacterium, the eCIS structures were shown to be anchored to the thylakoid membrane by a transmembrane helix in a fiber-like protein that was attached to the baseplate^13^. Although we did not observe fibers on the *Sco* eCIS particles, we did detect the putative fiber protein in our purified samples by mass spectrometry (Supplementary Table 1). The conditions of cell lysis during purification would likely disrupt membrane interactions leaving the fibers in variable conformations that might be difficult to distinguish by TEM.

Another mechanism of action for the *Sco* eCIS was suggested by Casu et al.^46^. Using cryoelectron tomography (cryoET), they observed free-floating extended eCIS exclusively within intact hyphae. In partially lysed hyphae, they saw a mixture of extended and contracted eCIS, while ghost cells, which are membrane-less cells displaying only a peptidoglycan layer, contained only contracted eCIS. They concluded that the eCIS action involving sheath contraction causes lysis from within the cells. Our data are also consistent with this model with the assumption that ActR does not escape readily from ghost cells or that only a small percentage of cells were lysed during the period of ActR induction in our experiments (45 min).

One potential limitation of our study is that some key experiments, such as those investigating cell death, were performed in liquid cultures in which *Sco* does not sporulate. However, the developmental program with respect to gene expression has been shown to be similar in liquid and solid medium^48,49^. Thus, we expect to see related differences during growth on solid media where cell death has also been shown to occur^50–52^.

Vegetative hyphal degradation during development has long been recognized as a feature of *Streptomycete* development^53^ and more recently programmed cell death has been postulated to occur as part of the shift from vegetative to aerial mycelial growth^25,50,54^. It is thought that a coordinated stage of cell death limited to a subset of the vegetative hyphal population during development may serve to provide additional nutrients to the growing reproductive aerial mycelium and dead hyphae may also serve a structural role or facilitate nutrient transport^51,52,55,56^. Consistent with a role at this stage in the *Sco* lifecycle, we have shown that eCIS expression peaks late in the vegetative growth phase in a temporal pattern similar to the antibiotic regulator gene, *redD*. The requirement of BldA, a master regulator of the morphogenetic switch, for the expression of the *Sco* eCIS genes also supports a role in this process. The apparent requirement for *bldA* for expression of eCIS genes in many *Streptomyces* species as well as the conservation of putative effector and fiber proteins among many *Streptomyces* eCIS regions suggest that these eCIS may share a conserved function in programmed cell death. This shared function in the *Streptomyces* lifecycle would explain the very frequent occurrence of eCIS in this clade. Interestingly, a role in intra-strain cell death was recently proposed for the eCIS of *Anabaena*, which also has a complex lifestyle^13^. We found that the eCIS of diverse Actinomycetes and other filamentous Cyanobacteria encode homologues of the Sco4256 putative effector protein, providing another connection between eCIS function in these two filamentous groups of bacteria.

To conclude, this study has revealed a function for eCIS in mediating intra-strain cell lethality that impacts the developmental process of *Streptomyces*, supporting that eCIS can possess bactericidal activity and participate in the normal lifecycle of a bacterial species.

## Methods

### Bacterial strains, plasmids, and media

Bacterial strains, plasmids and cosmids used in this study are listed in Supplementary Table 3. All *Streptomyces* strains used were derivatives of *Streptomyces coelicolor* A3(2). *Sco* strains were grown at 30 °C on maltose-yeast extract-malt extract (MYM) agar medium^57^ for 3 days or in yeast extract-malt extract (YEME) liquid medium^58^ with continuous agitation for 3 days, unless indicated otherwise. Other bacterial strains were grown in Lysogeny Broth (LB) medium or agar (pH 7.0) at 37 °C unless indicated otherwise. *Saccharomyces cerevisiae* were grown in YPD medium or agar (pH 7.0) at 30 °C.

### Construction and testing of Lux reporter strains

The eCIS promoter sequences were amplified from wild-type *Sco* M145 genomic DNA using primers SCO_eCISp1p2GEN and SCO_eCISp3p4GEN (Supplementary Table 4). The resulting PCR fragments were used as a template to introduce an EcoRV blunt cut site using SCO_eCISp1p2EcoRV and SCO_eCISp3p4EcoRV primers. The PCR products were cloned into the EcoRV site of the pF*lux* plasmid^32^ in the forward and the reverse orientation for the ability to test both transcriptional directions of the divergent promoter regions. All plasmids were verified by sequencing. The resulting reporter plasmids pF*lux-*eCISp1-p4 were electroporated into methylation-deficient *E. coli* ET12567/pUZ8002 cells^58^. Plasmids were introduced into wild-type *Sco* by intergenic conjugation and selection on apramycin (50 µg/mL)^58^.

For luminescence assays 10^4^ spore forming units (SFU) of each *Sco* reporter strain were resuspended in 10 µL of saline and plated in triplicate onto 200 μl of soya-mannitol (MS) agar^59^ in 96-well polystyrene plates. Plates were incubated for 7 days at 30 °C and luminescence was measured three times daily using a PerkinElmer Victor X multilabel plate reader.

### Generation of gene replacement mutants

To create genomic replacements of the eCIS tail sheath, baseplate and ATPase encoding genes, the REDIRECT method for PCR targeting in *Streptomyces* was employed as described^58^. The *aac3(IV)-oriT* cassette, which confers resistance to apramycin, was amplified using SCO4253_Disrp, SCO_BP_Disrp or SCO4259_Disrp primer pairs, respectively (Supplementary Table 4). The amplified cassettes were used to replace the respective eCIS genes in cosmid StD8a^60^. The resulting cosmids (listed in Supplementary Table 3) were introduced by conjugation from the non-methylating *E. coli* strain ET12567/pUZ8002^58^ into the wild-type strain M145 to obtain apramycin-resistant exconjugants. To select for double cross-over exconjugants the aparamycin resistant colonies were screened for kanamycin resistance. Three Apra^R^ Kan^S^ exconjugant strains were selected for each gene, resulting from separate conjugation events. Mutant strains were confirmed by PCR using oligonucleotides; StD8aΔTS_aac(3)IV, StD8aΔBP_aac(3)IV or StD8aΔATP_aac(3)IV (Supplementary Table 4), respectively and by DNA sequencing.

### Actinorhodin production assay

Wild-type *Sco* M145 or *Sco* Δ*sco4253* (ΔTS) were inoculated in experimental triplicate into R2YE liquid media at a final concentration of 1.5 × 10^6^ SFU/mL. Cultures were grown with agitation for 22 hr at 30 °C. All cultures were standardized to an OD_450_ of 0.5. Cells were recovered by centrifugation at 3500 × *g* for 10 min, resuspended in saline and centrifuged again at 3500 × *g* for 10 min. Cell pellets were resuspended in 50 mL of fresh RG2 minimal media. The cultures were incubated at 30 °C with continuous shaking for 94 hr. For visual comparison of pigment production, images of the growing cultures were taken using a NIKON D3000 camera at a fixed position within a light box at times of 0, 18 hr, 24 hr, 41 hr, 49 hr, 65 hr, 70 hr and 94 hr. For quantification of total actinorhodin production, 480 µl samples were collected at the time points indicated above. 120 µl of 5 M KOH was added to a final concentration of 1 M, and samples were vortexed and centrifuged at 3000× *g* for 5 min. The absorbance of the supernatant at 640 nm was read using a polystyrene 96 well plate. Each assay was standardized to the weight of the pellet.

### Aerial mycelium formation assay

10^8^ SFU of *Sco* wild-type or eCIS-deficient mutant strains in biological duplicate were plated on R2YE agar plates and grown at 30 °C. Starting from 24 hr postgermination, sterile glass cover slips were gently applied to the top surface of each bacterial lawn at approximately 12 hr intervals. Cover slips were sealed on top of a microscope glass slide and examined using a Zeiss-transmitted light microscope at 40X magnification. Images were subsequently analyzed using Adobe Photoshop CS6 v.13.0. The background was normalized to an empty WT slide and the total pixel density was calculated for each image. Two sample t-Test assuming equal variance was performed to compare the mean hyphal density of each eCIS mutant to the WT sample mean.

### Inter-species growth inhibition assays

Spores (10^5^ SFU suspended in 5 µL of 0.85% saline) of wild-type *Sco* M145, M1152 or the eCIS mutant strains ΔTS or M1152 ΔTS were spotted on R2YE agar plates. Plates were incubated for 3 days at 30°C before being overlaid with 5 mL molten, extra-soft LB agar (0.5% agar) containing organisms to be tested (at a final OD_600_ ranging from 0.01–0.1). Plates were incubated at 37 °C overnight and antimicrobial activity was assessed by measurement of zones of clearance in the indicator lawn. The surface area of the zones of clearance induced by *Sco* M145 strains was quantified using ImageJ v. 1.53. Assays against *Streptomyces* strains were conducted by overlaying a 3-day colony plate with 1 mL 0.85% saline solution containing 5 µL of concentrated *Streptomyces* spores. Plates were incubated for 3 days at 30 °C before antimicrobial activity was assessed. Assays against *Saccharomyces cerevisiae* were conducted by overlaying a 4-day colony plate with 5 mL molten, extra-soft YPD agar containing *S. cerevisiae* (at a final OD_600_ ranging from 0.05–0.2). Plates were incubated at 30 °C overnight.

### Generation of antibodies against TS and BW1 proteins

The TS and BW1 proteins were expressed and purified from *E. coli*. Genes encoding the BW1 protein (*sco4245*) and the TS protein (*sco4253*) were cloned from *Sco* genomic DNA into the BseR1 site of p15TV-L vector using the In-fusion HD cloning kit (Clonetech) using primers listed in Supplementary Table 4. Verified plasmids were transformed into *E. coli* strain BL21 (DE3), grown at 37 °C with appropriate antibiotics to an OD_600_ of 0.6 and protein expression was induced with 1 mM IPTG. Cultures were grown for 16–18 hr at 17 °C. Cells were collected by centrifugation and resuspended in binding buffer (20 mM Tris-HCl pH 7.5, 200 mM NaCl, 5 mM imidazole, 5 mM β-mercaptoethanol (βME)). Cells were lysed by sonication. Lysates were cleared by centrifugation at 25,000 × *g* for 20 minutes at 4 °C. Proteins were affinity-purified by incubation with 2 mL of Ni-NTA agarose resin (Invitrogen) for 30 min at 4 °C. The mixture was passed through a column at room temperature and washed extensively with binding buffer containing 30 mM imidazole. Bound proteins were eluted with binding buffer containing 300 mM imidazole and dialyzed overnight at 4 °C in buffer containing; 20 mM Tris·HCl, pH 7.5, 200 mM NaCl, 5% (vol/vol) glycerol, 1 mM DTT. Samples were concentrated using Vivaspin filter concentrators (Sigma-Aldrich) and further separated using Superdex200 16/60 size exclusion column on an ÄKTA fast performance liquid-chromatography system (GE Healthcare). Relevant fractions were analyzed and verified by SDS-PAGE and concentrated to a final concentration of 0.1 – 0.25 mg/ml.

Mouse polyclonal antibodies were generously generated by the Gray-Owen lab at the University of Toronto. Fresh purified protein at a concentration of 0.05–0.25 mg/mL was mixed with 100 µl Emulsigen adjuvant and injected into mice. A second booster dose was administered 21 days later. Antisera were collected on day 35. Antisera specificity was tested by western blot analysis with appropriate controls. Source data and validation are provided within the Source Data file.

### eCIS particle purification

*Sco* strains were grown on MYM agar at 30 °C for 4 days. 1 cm^2^ squares of agar containing mycelium and spores were excised from the plate and directly transferred into 50 mL YEME liquid medium. Cells were incubated at 30 °C for the indicated various times with shaking. Cells were pelleted and extracellular sample (E) was taken from the supernatant. Pellets were washed in 10% sucrose and centrifuged again, resuspended in 16 mL of buffer P^61^ supplemented with 2 mg/mL lysozyme and incubated at 30 °C for 1 hr. Cells were spun down and resuspended in Buffer C (50 mM Tris pH 7.5, 150 mM NaCl, 15% vol/vol glycerol, 1 mM DTT, protease inhibitor cocktail (Sigma-Aldrich-one tablet per 50 mL of buffer)), and sonicated. Lysed cells were centrifuged at 12,000 × *g* for 20 minutes and the supernatant was then filtered through a 0.45 µm filter. Filtered samples were spun at 150,000 × *g* for 3 h at 4 °C. The pellet was resuspended in 5 mL of ice cold 1XPBS. The samples were loaded onto columns containing 5 mL of DEAE Sepharose anion exchange resin (Sigma-Aldrich), washed extensively with PBS and eluted using 0.5 M NaCl in PBS buffer. Samples were ultracentrifuged at 150,000 × *g* for 90 min at 4 °C and pellets were resuspended in 200 μL ice-cold PBS and stored at 4 °C for up to two weeks.

### Western blot assay

Samples were mixed in a 1:1 ratio with 2 X SDS loading buffer (100 mM Tris-HCl, pH 6.8; 4% SDS; 20% glycerol; 200 mM β-mercaptoethanol (βME); 0.2% Bromophenol blue). Proteins were separated on a 12% polyacrylamide SDS Tris-Tricine gels and transferred to a nitrocellulose membrane. The membrane was blocked for 1 hr in 5% skim milk in Tris-buffered saline with 1% Tween (TBS-T). When tested with α-eCIS specific antibodies, antiserum was added at a 1:5000 dilution in TBS-T with 5% milk. Alternatively, α-FLAG primary antibody (Sigma-Aldrich, F7425) was added at a 1:10,000 dilution. Membranes were incubated overnight at 4 °C. After washing, the membranes were probed with horseradish peroxidase-conjugated goat antimouse (IgG-HRP sc-2005, Santa Cruz Biotechnology) or mouse anti-rabbit (IgG HRP sc-2357, Santa Cruz Biotechnology) at a 1:2000 dilution in TBS-T for 1 hr at room temperature and developed using chemiluminescent reagents (GE Healthcare).

### Transmission electron microscopy

Purified eCIS samples were briefly centrifuged to remove impurities and 5 μL of supernatant was applied onto freshly glow-discharged carbon-coated copper grids (CF400-CU, 6 nm, 400 mesh, Electron Microscopy Sciences). Samples were allowed to adsorb for 2 minutes before being blotted off by filter paper and grids were washed twice with filtered distilled water. The grids were negatively stained with 15 μL of 2% uranyl acetate for 15 seconds. Grids were imaged with a JEM-1011 (JEOL USA, INC.), digital CDD camera (5 megapixels XR50S, AMT, USA).

### Live/Dead assay

*Sco* wild-type or eCIS mutant strains were inoculated into 40 mL of YEME culture medium from fresh spores at a density of 10^7^ /mL and incubated at 30 °C with shaking. Samples of 1 mL were centrifuged for 5 min at 8000 × *g*, washed twice and resuspended in 1 mL of distilled water. LIVE/DEAD Bac-Light bacterial viability kit (L7012; Invitrogen) was used to stain cells. The bacterial suspension was mixed with 3 µl of SYTO 9 and propidium iodide (PI) nucleic acid stains, premixed at a 1:1 ratio. The cell suspension and nucleic acid stains were mixed by vortex and left to stand at room temperature for 10 min in the dark. 10 µl of the suspension was deposited on a clean slide and covered with an 18 mm square coverslip. Images were acquired within 30 min of incubation using a Zeiss Axio imager 2, at an excitation of 488 nm and 568 nm and emission of 530 nm (green) or 630 nm (red). Images were analyzed using Zeiss Zen Blue, version 3.0.19154.1 (Carl Zeiss Microscopy GmbH) and statistical analysis was performed using GraphPad Prism 9 version 9.5.0.

### Mass spectrometry analysis of purified *Sco* eCIS samples

eCIS samples were purified as described above. Extracellular samples were similarly purified from cell-free supernatants following the initial centrifugation step at 7000 × *g*. Purified samples of 30–150 µg total protein were reduced with DTT, alkylated with iodoacetamide, and subjected to tryptic digest. Liquid chromatography tandem-mass spectrometry spectra were collected on a linear ion-trap instrument (Thermo Fisher) (SPARC BioCentre, The Hospital for Sick Children, Toronto, Canada). Proteins were identified using Mascot (Matrix Science, London, UK) and analyzed in Scaffold version 5.2.2 (Proteome Software Inc., Portland, OR, USA). The cut off for protein identification was set at a confidence level of 95%.

### Reporting summary

Further information on research design is available in the Nature Portfolio Reporting Summary linked to this article.

## Supplementary information

Supplementary Information

Description of Additional Supplementary Files

Supplementary Dataset 1-7

Reporting Summary

Peer Review File

## Data Availability

The mass spectrometry data for purified *Sco* eCIS samples generated in this study has been deposited in MassIVE database under accession code; MSV000091288 [10.25345/C5CC0V388]. All remaining data are available in the Supplementary Information, Supplementary Data 1-7 and the Source Data file. Source data are provided with this paper.

## Source data

Source Data

## References

[1] Taylor NMI, van Raaij MJ, Leiman PG (2018). Contractile injection systems of bacteriophages and related systems. Mol. Microbiol.

[2] Geller AM (2021). The extracellular contractile injection system is enriched in environmental microbes and associates with numerous toxins. Nat. Commun..

[3] Chen L (2019). Genome-wide Identification and Characterization of a Superfamily of Bacterial Extracellular Contractile Injection Systems. Cell Rep..

[4] Rybakova D (2013). Role of antifeeding prophage (Afp) protein Afp16 in terminating the length of the Afp tailocin and stabilizing its sheath. Mol. Microbiol.

[5] Jiang F (2019). Cryo-EM Structure and Assembly of an Extracellular Contractile Injection System. Cell.

[6] Zhang D, de Souza RF, Anantharaman V, Iyer LM, Aravind L (2012). Polymorphic toxin systems: Comprehensive characterization of trafficking modes, processing, mechanisms of action, immunity and ecology using comparative genomics. Biol. Direct.

[7] Makarova, K. S. et al. Antimicrobial Peptides, Polymorphic Toxins, and Self-Nonself Recognition Systems in Archaea: an Untapped Armory for Intermicrobial Conflicts. *mBio* **10**, 10.1128/mBio.00715-19 (2019). 10.1128/mBio.00715-19 PMC6509191 31064832

[8] Hurst MR, Glare TR, Jackson TA (2004). Cloning Serratia entomophila antifeeding genes–a putative defective prophage active against the grass grub Costelytra zealandica. J. Bacteriol..

[9] Yang G, Dowling AJ, Gerike U, ffrench-Constant RH, Waterfield NR (2006). Photorhabdus virulence cassettes confer injectable insecticidal activity against the wax moth. J. Bacteriol..

[10] Vlisidou, I. et al. The Photorhabdus asymbiotica virulence cassettes deliver protein effectors directly into target eukaryotic cells. *Elife* **8**, 10.7554/eLife.46259 (2019). 10.7554/eLife.46259 PMC6748792 31526474

[11] Shikuma NJ (2014). Marine tubeworm metamorphosis induced by arrays of bacterial phage tail-like structures. Science.

[12] Bock D (2017). In situ architecture, function, and evolution of a contractile injection system. Science.

[13] Weiss GL (2022). Structure of a thylakoid-anchored contractile injection system in multicellular cyanobacteria. Nat. Microbiol.

[14] Xu J (2022). Identification and structure of an extracellular contractile injection system from the marine bacterium Algoriphagus machipongonensis. Nat. Microbiol.

[15] Chater KF (1972). A morphological and genetic mapping study of white colony mutants of Streptomyces coelicolor. J. Gen. Microbiol.

[16] Marie A. Elliot., M. J. B., Justin R. *Nodwell*. In Myxobacteria: Multicellularity and Differentiation (ed y David E. Whitworth) 419–439 (ASM Press, 2008).

[17] Flardh K, Buttner MJ (2009). Streptomyces morphogenetics: dissecting differentiation in a filamentous bacterium. Nat. Rev. Microbiol.

[18] Buttner CR, Wu Y, Maxwell KL, Davidson AR (2016). Baseplate assembly of phage Mu: Defining the conserved core components of contractile-tailed phages and related bacterial systems. Proc. Natl Acad. Sci. USA.

[19] Hurst MR, Beard SS, Jackson TA, Jones SM (2007). Isolation and characterization of the Serratia entomophila antifeeding prophage. FEMS Microbiol Lett..

[20] Desfosses A (2019). Atomic structures of an entire contractile injection system in both the extended and contracted states. Nat. Microbiol.

[21] Heymann JB (2013). Three-dimensional structure of the toxin-delivery particle antifeeding prophage of Serratia entomophila. J. Biol. Chem..

[22] Nagakubo T (2021). Phage tail-like nanostructures affect microbial interactions between Streptomyces and fungi. Sci. Rep..

[23] Gomez-Escribano JP, Bibb MJ (2011). Engineering Streptomyces coelicolor for heterologous expression of secondary metabolite gene clusters. Micro. Biotechnol..

[24] Boulos L, Prevost M, Barbeau B, Coallier J, Desjardins R (1999). LIVE/DEAD BacLight: application of a new rapid staining method for direct enumeration of viable and total bacteria in drinking water. J. Microbiol Methods.

[25] Manteca A, Alvarez R, Salazar N, Yague P, Sanchez J (2008). Mycelium differentiation and antibiotic production in submerged cultures of Streptomyces coelicolor. Appl Environ. Microbiol.

[26] Yague P, Manteca A, Simon A, Diaz-Garcia ME, Sanchez J (2010). New method for monitoring programmed cell death and differentiation in submerged Streptomyces cultures. Appl Environ. Microbiol.

[27] Fernandez-Moreno MA, Caballero JL, Hopwood DA, Malpartida F (1991). The act cluster contains regulatory and antibiotic export genes, direct targets for translational control by the bldA tRNA gene of Streptomyces. Cell.

[28] Floriano B, Bibb M (1996). afsR is a pleiotropic but conditionally required regulatory gene for antibiotic production in Streptomyces coelicolor A3(2). Mol. Microbiol.

[29] Aceti DJ, Champness WC (1998). Transcriptional regulation of Streptomyces coelicolor pathway-specific antibiotic regulators by the absA and absB loci. J. Bacteriol..

[30] Gao, C., Hindra, Mulder, D., Yin, C. & Elliot, M. A. Crp is a global regulator of antibiotic production in streptomyces. *mBio* **3**, 10.1128/mBio.00407-12 (2012). 10.1128/mBio.00407-12 PMC3520106 23232715

[31] Xu, Z., Li, Y., Wang, Y., Deng, Z. & Tao, M. Genome-Wide Mutagenesis Links Multiple Metabolic Pathways with Actinorhodin Production in Streptomyces coelicolor. *Appl. Environ. Microbiol.* **85**, 10.1128/AEM.03005-18 (2019). 10.1128/AEM.03005-18 PMC6585502 30709825

[32] Craney A (2007). A synthetic luxCDABE gene cluster optimized for expression in high-GC bacteria. Nucleic Acids Res..

[33] Shiina T, Tanaka K, Takahashi H (1991). Sequence of hrdB, an essential gene encoding sigma-like transcription factor of Streptomyces coelicolor A3(2): homology to principal sigma factors. Gene.

[34] Sun J, Kelemen GH, Fernandez-Abalos JM, Bibb MJ (1999). Green fluorescent protein as a reporter for spatial and temporal gene expression in Streptomyces coelicolor A3(2). Microbiol. (Read.).

[35] Zhou LH, Li YQ, Li YQ, Wu D (2005). Spatio-temporal expression of the pathway-specific regulatory gene redD in S. coelicolor. J. Zhejiang Univ. Sci. B.

[36] Hackl S, Bechthold A (2015). The Gene bldA, a regulator of morphological differentiation and antibiotic production in streptomyces. Arch. Pharm. (Weinh.).

[37] Hesketh A (2007). New pleiotropic effects of eliminating a rare tRNA from Streptomyces coelicolor, revealed by combined proteomic and transcriptomic analysis of liquid cultures. BMC Genom..

[38] Kim DW, Chater KF, Lee KJ, Hesketh A (2005). Effects of growth phase and the developmentally significant bldA-specified tRNA on the membrane-associated proteome of Streptomyces coelicolor. Microbiol. (Read.).

[39] Chandra G, Chater KF (2008). Evolutionary flux of potentially bldA-dependent Streptomyces genes containing the rare leucine codon TTA. Antonie Van. Leeuwenhoek.

[40] Guyet A (2014). Identified members of the Streptomyces lividans AdpA regulon involved in differentiation and secondary metabolism. BMC Microbiol.

[41] White J, Bibb M (1997). bldA dependence of undecylprodigiosin production in Streptomyces coelicolor A3(2) involves a pathway-specific regulatory cascade. J. Bacteriol..

[42] Ericson, C. F. et al. A contractile injection system stimulates tubeworm metamorphosis by translocating a proteinaceous effector. *Elife* **8**, 10.7554/eLife.46845 (2019). 10.7554/eLife.46845 PMC6748791 31526475

[43] Rocchi I (2019). A Bacterial Phage Tail-like Structure Kills Eukaryotic Cells by Injecting a Nuclease Effector. Cell Rep..

[44] Sayers EW (2021). Database resources of the National Center for Biotechnology Information. Nucleic Acids Res..

[45] Soding J, Biegert A, Lupas AN (2005). The HHpred interactive server for protein homology detection and structure prediction. Nucleic Acids Res.

[46] Casu, B., Sallmen, J. W., Schlimpert, S. & Pilhofer, M. Cytoplasmic contractile injection systems mediate cell death in *Streptomyces*. *bioRxiv*, 2022.2008.2009.503279, 10.1101/2022.08.09.503279 (2022). 10.1038/s41564-023-01341-x PMC10066040 36894633

[47] Habann M (2014). Listeria phage A511, a model for the contractile tail machineries of SPO1-related bacteriophages. Mol. Microbiol.

[48] Yague P (2014). Transcriptomic analysis of liquid non-sporulating Streptomyces coelicolor cultures demonstrates the existence of a complex differentiation comparable to that occurring in solid sporulating cultures. PLoS One.

[49] Manteca A, Jung HR, Schwammle V, Jensen ON, Sanchez J (2010). Quantitative proteome analysis of Streptomyces coelicolor Nonsporulating liquid cultures demonstrates a complex differentiation process comparable to that occurring in sporulating solid cultures. J. Proteome Res..

[50] Tenconi E, Traxler MF, Hoebreck C, van Wezel GP, Rigali S (2018). Production of Prodiginines Is Part of a Programmed Cell Death Process in Streptomyces coelicolor. Front Microbiol.

[51] Miguelez EM, Hardisson C, Manzanal MB (1999). Hyphal death during colony development in Streptomyces antibioticus: morphological evidence for the existence of a process of cell deletion in a multicellular prokaryote. J. Cell Biol..

[52] Manteca A, Fernandez M, Sanchez J (2006). Cytological and biochemical evidence for an early cell dismantling event in surface cultures of Streptomyces antibioticus. Res Microbiol.

[53] Wildermuth H (1970). Development and organization of the aerial mycelium in Streptomyces coelicolor. J. Gen. Microbiol.

[54] Filippova SN, Vinogradova KA (2017). Programmed cell death as one of the stages of streptomycete differentiation. Microbiology.

[55] Mendez C, Brana AF, Manzanal MB, Hardisson C (1985). Role of substrate mycelium in colony development in Streptomyces. Can. J. Microbiol.

[56] Chater KF (2006). Streptomyces inside-out: a new perspective on the bacteria that provide us with antibiotics. Philos. Trans. R. Soc. Lond. B Biol. Sci..

[57] Bibb MJ, Domonkos A, Chandra G, Buttner MJ (2012). Expression of the chaplin and rodlin hydrophobic sheath proteins in Streptomyces venezuelae is controlled by sigma(BldN) and a cognate anti-sigma factor, RsbN. Mol. Microbiol.

[58] Gust, B., Challis, G. L., Fowler, K., Kieser, T. & Chater, K. F. PCR-targeted *Streptomyces* gene replacement identifies a protein domain needed for biosynthesis of the sesquiterpene soil odor geosmin. *Proc. Natl. Acad. Sci. USA* **100**, 1541–1546 (2003). 10.1073/pnas.0337542100 PMC149868 12563033

[59] Hobbs G, Frazer CM, Gardner DCJ, Cullum JA, Oliver SG (1989). Dispersed growth of Streptomyces in liquid culture. Appl. Microbiol. Biotechnol..

[60] Redenbach M (1996). A set of ordered cosmids and a detailed genetic and physical map for the 8 Mb Streptomyces coelicolor A3(2) chromosome. Mol. Microbiol.

[61] Kieser, T. et al. *Practical Streptomyces genetics*. (John Innes Foundation, 2000).

[62] Edgar RC (2004). MUSCLE: multiple sequence alignment with high accuracy and high throughput. Nucleic Acids Res..

[63] Waterhouse AM, Procter JB, Martin DM, Clamp M, Barton GJ (2009). Jalview Version 2–a multiple sequence alignment editor and analysis workbench. Bioinformatics.

