# Supplementary material for: A contractile injection system is required for developmentally regulated cell death in Streptomyces coelicolor
Source: Nat Commun. 2023 Mar 16;14:1469. doi: 10.1038/s41467-023-37087-7 (PMC10020575; doi:10.1038/s41467-023-37087-7)
Supplement: Supplementary file 1 — Supplementary Information [file 41467_2023_37087_MOESM1_ESM.pdf]

Supplementary Information

# A contractile injection system is required for developmentally regulated cell death in *Streptomyces coelicolor*

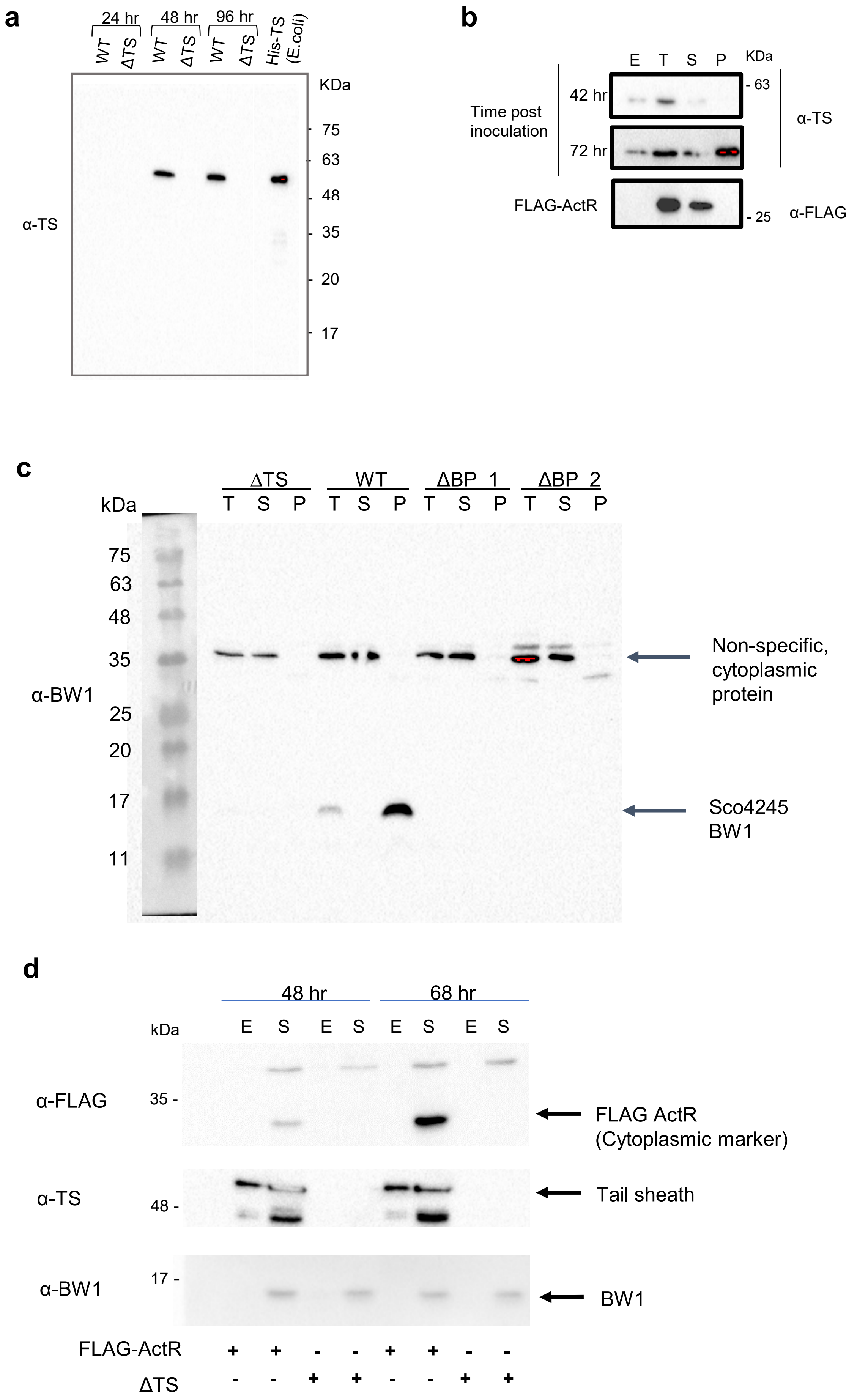

a
24 hr
48 hr
96 hr
WT
ΔTS
His-TS (E.coli)
KDa
75
63
48
35
20
17
α-TS
b
E T S P
KDa
Time post inoculation
42 hr
72 hr
- 63
α-TS
FLAG-ActR
- 25
α-FLAG
c
ΔTS
WT
ΔBP_1
ΔBP_2
T S P
kDa
75
63
48
35
25
20
17
11
α-BW1
Non-specific, cytoplasmic protein
Sco4245 BW1
d
48 hr
68 hr
E S E S E S E S
kDa
α-FLAG
35 -
FLAG ActR (Cytoplasmic marker)
α-TS
48 -
Tail sheath
α-BW1
17 -
BW1
FLAG-ActR + + - - + + - -
ΔTS - - + + - - + +

**Supplementary Fig. 1| eCIS proteins are detected by specific polyclonal antibodies in lysates of WT *Sco*. a,** *Sco* wild-type (WT) or a tail sheath knockout strain (ΔTS) were grown in liquid R2YE media to the indicated time points. Whole cell lysates were analyzed by Western blot using α-TS sera. Heterologously expressed and purified 6XHis-tagged-tail sheath (His-TS) was used as a positive control. **b,** WT *Sco* cells were grown in liquid YEME media, cells were collected at the indicated time points and an extracellular "E" sample was taken from the cell-free supernatant. Cells were lysed and an intracellular total lysate "T" sample was collected. The lysate was ultracentrifuged for 3 hr at 150,000 x g, the supernatant "S" and pellet "P" were collected. Samples were probed using α-TS antibodies. For the *Sco* strain expressing FLAG-tagged ActR from a plasmid, expression was induced with 30 µg/mL final concentration of thiostrepton, added 45 minutes before cells were harvested by centrifugation. **c,** *Sco* wild-type (WT) or a baseplate knockout (ΔBP) were grown and fractionated as in (b). Samples were probed by Western blot using α-BW1 antibodies. **d,** *Sco* expressing FLAG-ActR or a tail sheath knockout strain (ΔTS) were grown for 48 or 68 hr and separated as in (b). Extracellular "E" and lysate "S" samples were probed by Western blot using α-TS, α-BW1 or α-FLAG antibodies. Western blots show representative results from three independent fractionation experiments. Source unprocessed blots are provided within the Source Data file.

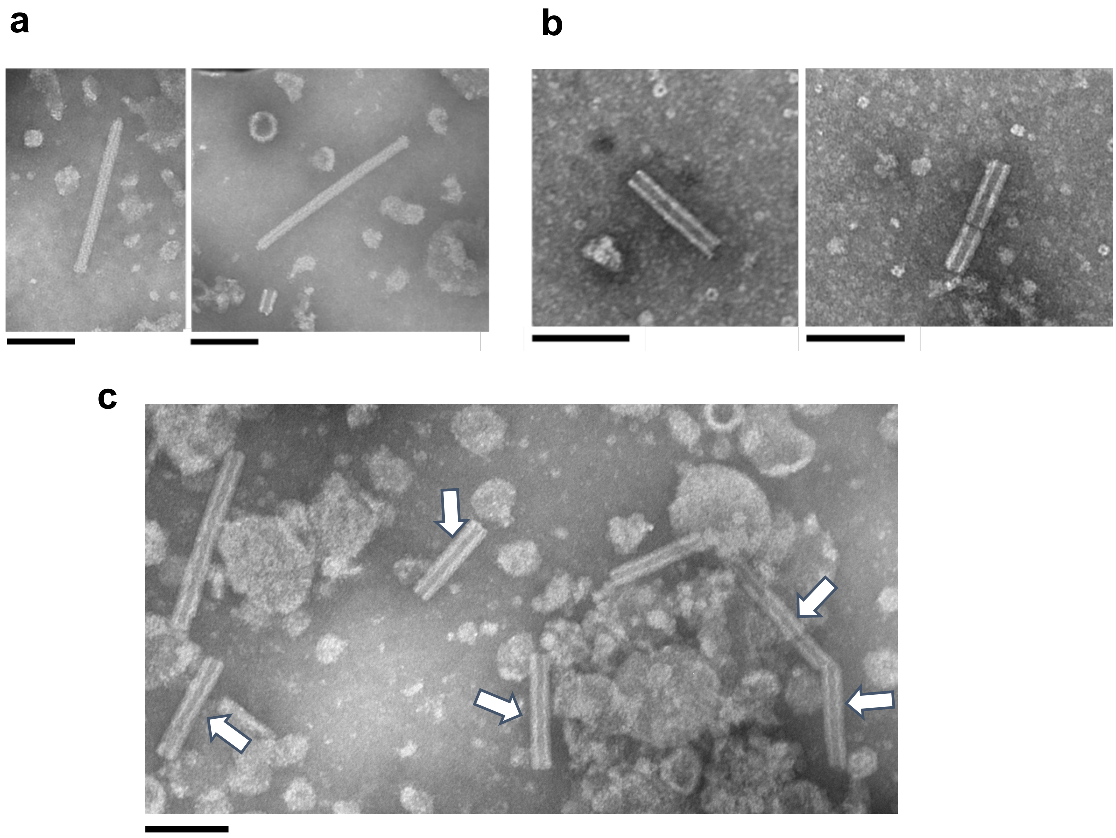


**Supplementary Fig. 2| The *Sco* ATPase mutant produces both mature eCIS and empty sheath particles, detectable in the lysate and extracellular media.** All images represent *Sco* ΔATPase grown in YEME liquid media for 48 hr (a), 54 hr (b) or 72 hr (c) post inoculation. **a,** Representative images of purified eCIS particles from cell lysate concentrated to 30X original culture after 48 hr of growth. The majority of particles at this time are in their extended conformation. **b,** The extracellular fraction after 54 hr of growth is shown. White arrows point to typical emptied contracted tail sheath particles. **c,** Purified lysate from cells grown for 72 hr. Emptied sheath particles are indicated with white arrows. Scale bar = 100 nm. The micrographs shown are representative of results obtained for three separate biological replicates.

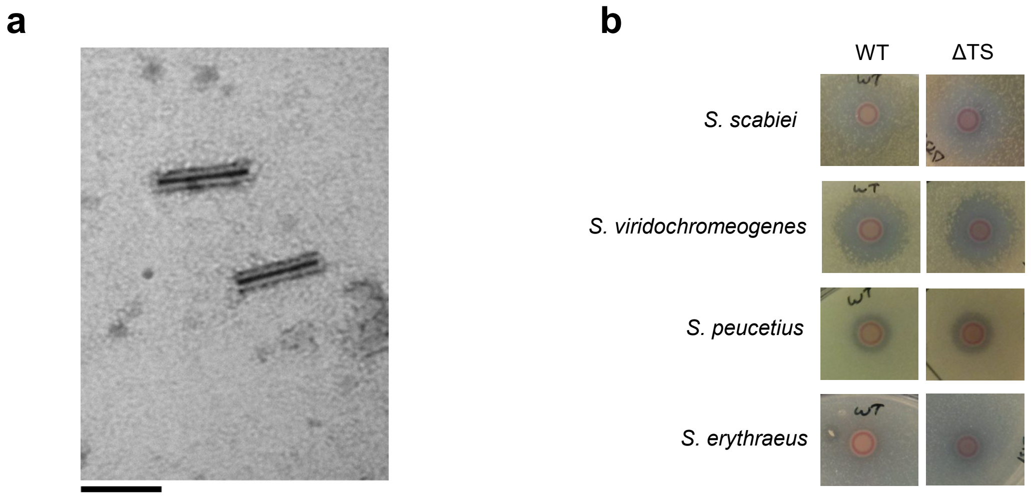


**Supplementary Fig. 3| Growth inhibition of other *Streptomyces* species by WT and eCIS-deficient *Sco* strains. a, A** representative TEM image of cell-free media collected from *Sco* M1152 strain grown for 72 hr. Empty contracted sheath particles, visually identical to ones produced by *Sco* WT M145 strain are observed. The presence of these particles is indicative of production of normal eCIS particles that can contract. No particles were detected in a M1152 strain harboring a tail sheath knockout mutation (M1152 ΔTS). Scale bar = 100 nm. **b,** Assays were performed by spotting on agar plates $10^5$ SFU of *Sco* wild-type (WT) or tail sheath knockout (ΔTS) strains. Cells were grown for 3 days before being overlaid with species to be tested as indicated in each panel. Zones of clearing around the *Sco* colonies indicate lethality or growth inhibitory activity against the indicator lawn. The results shown for a tested species strain were obtained from the same agar plate. Images are representative examples of many replicates (n > 3).

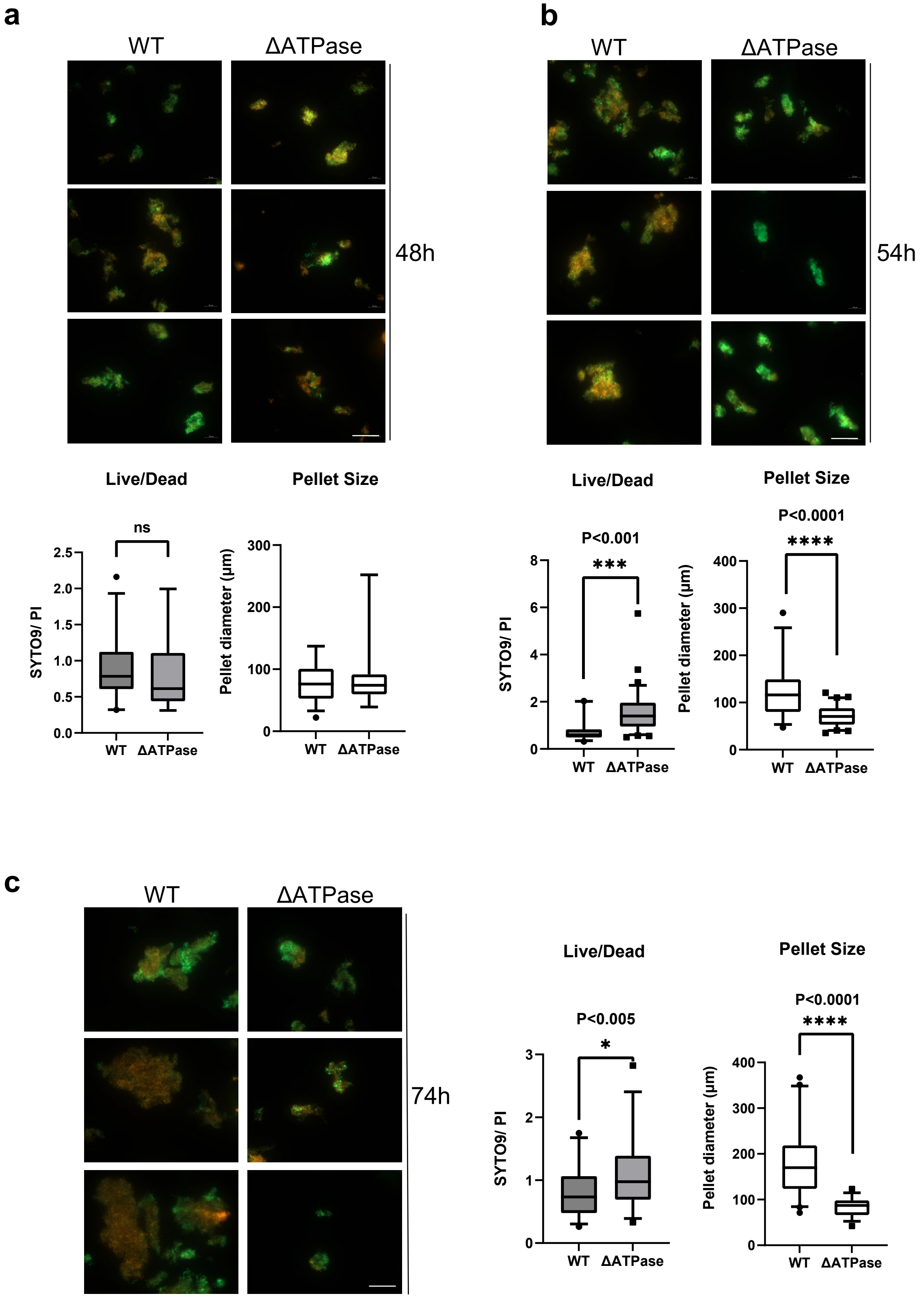
a
WT
ΔATPase
48h
Live/Dead
Pellet Size
ns
SYTO9/ PI
2.5
2.0
1.5
1.0
0.5
0.0
WT
ΔATPase
Pellet diameter (μm)
300
200
100
0
WT
ΔATPase
b
WT
ΔATPase
54h
Live/Dead
Pellet Size
P<0.001
***
SYTO9/ PI
8
6
4
2
0
WT
ΔATPase
P<0.0001
****
Pellet diameter (μm)
400
300
200
100
0
WT
ΔATPase
c
WT
ΔATPase
74h
Live/Dead
Pellet Size
P<0.005
*
SYTO9/ PI
3
2
1
0
WT
ΔATPase
P<0.0001
****
Pellet diameter (μm)
400
300
200
100
0
WT
ΔATPase

**Supplementary Fig. 4| The *Sco* ATPase deletion mutant strain exhibits reduced cell death during hyphal differentiation. a-c,** *Sco* wild-type (WT) or an ATPase knockout (ΔATPase) strains were inoculated into YEME media at a final concentration of $10^7$ SFU /mL. At 48 hr (**a**), 54 hr (**b**) or 74 hr (**c**) post-inoculation, samples were stained with equal proportions of propidium iodide and SYTO 9. Total (live and dead) cells are stained with SYTO 9 and are visualized by green fluorescence. Cells with damaged membranes are stained only with PI and are visualized by red fluorescence. The ratio of SYTO 9 to PI (Live/dead ratio) was calculated from total fluorescence intensity values per image. Diameters of individual hyphal pellets were manually measured from each collected image (for WT and ΔATPase, respectively; at 48 hr – n=37, 29, at 54 hr – n=37, 70, at 74 hr – n=28, 33). Results are shown as box plots. The center horizontal line denotes the median and boxes extend from the $25^{th}$ to the $75^{th}$ percentile of values. Whiskers denote values within the $5\text{-}95^{th}$ percentile range, with individual data points shown for the values outside that range. Single factor ANOVA and Welch's t-test were performed to calculate statistical significance. Adjusted two-sided *P*-values are shown above the relevant graphs. In (b), *P*-value for live/dead ratios = 4.9E-04, *P*-value for pellet size =5.5E-10 . In (c), *P*-value for live/dead ratios = 0.0226 , *P*-value for pellet size = 9.5E-11. Scale bar = 100 µm.
Source data are provided as a Source Data file.

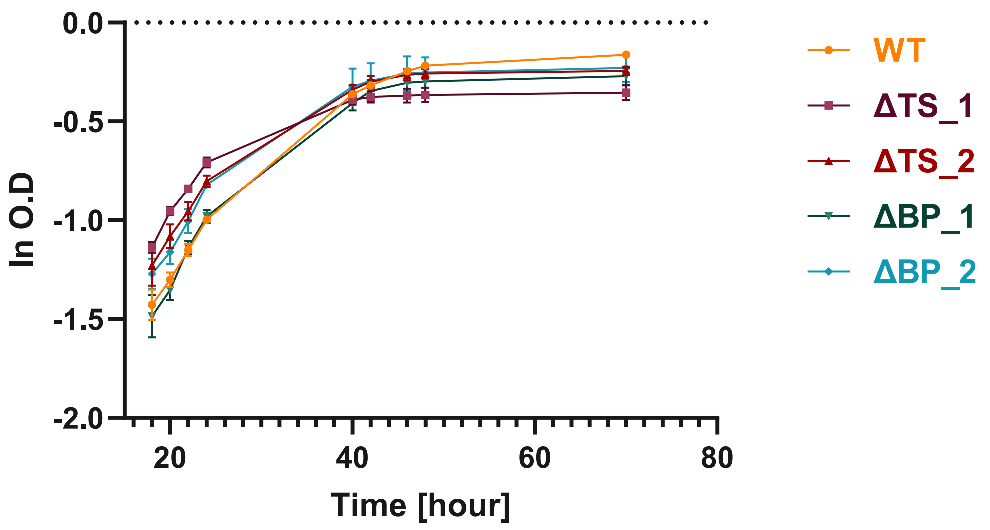


**Supplementary Fig. 5| Growth curve for WT *Sco* and eCIS-deficient mutant strains.** $10^8$ SFU of *Sco* WT, tail sheath knockout (ΔTS) or baseplate knockout (ΔBP) were inoculated in R2YE liquid media and grown for 70 hours. Optical density ($OD_{600}$) was taken at the indicated time points. ΔTS_1, ΔTS_2 and ΔBP_1, ΔBP_2 represent two biological replicates of each mutant. The mean is shown with error bars representing standard error of the mean (n=3).

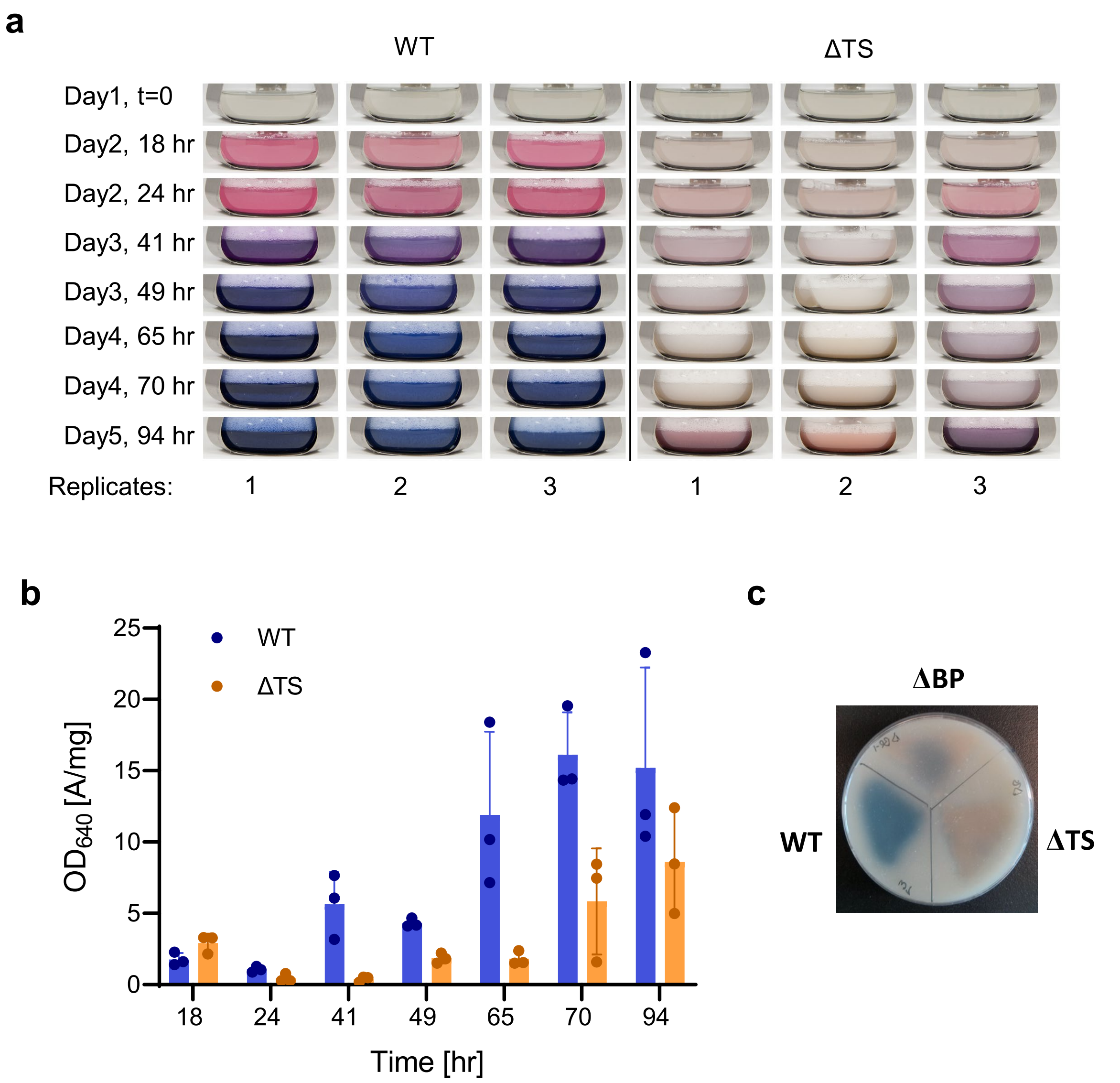


**Supplementary Fig. 6| eCIS mutants exhibit decreased actinorhodin production in minimal media. a,** Comparison of wild-type *Sco* (left) and ΔTS mutant (right) development in liquid media. Three experimental replicates of RG2 liquid media cultures were inoculated to an $OD_{450}$ of 0.5 and grown for 94 hours. Images of each culture flask were taken at the indicated time points. **b,** For quantification of total actinorhodin, samples were taken at the same time points as in (a). KOH was added to a final concentration of 1 M to extract the actinorhodin and the sample was centrifuged. The optical density ($OD_{640}$) of the supernatant was measured and normalized to wet pellet weight. The mean is shown with error bars representing standard deviation of the mean (n=3). **c,** Equal amounts of *Sco* wild-type, ΔTS or ΔBP spores were streaked out on minimal Soy Mannitol (MS) agar plates and grown for 4 days. The agar plate shown is representative of multiple independent assays with 3 biological replicates (strains ΔTS 1-3 , ΔBP 1-3, each obtained by a separate conjugation event).

## Promoter region *sco4248-49*

```
gatccacttggcgaccttcgccgtgtccggggtgagggggcgggtcagccggatgttgga
gtaggtgacgcgggtgggcagctgccagacgaagccgttgttgccgccctcctggcgctg
ttccacctccacctgggtcgacaggccctcgcacccgttgaagtagccgaggctctcgcc
gtcgatggcgaggctgaaccagatggaagagcccgggtccttacgggtcatcgggggtcg
acctttcttgtcggggagcggtggcggcgggcttggccctcggtgcggtccggccgtccg
gcggggccggtgcgggtccggtcagcgcgggtcgcgcagtctgccgatccgttcgcggtc
catacgcagttcggtgcggacgagacgggtgacgcggccgatgatccggtgcaccagttc
gtcgagctggaagtcggtgagttgacgcgggtcgaacccgccctcggggaccgccgtgta
cgcgggcgggggaccgtcgggggcggtgcgcccgcgcccgcctcgtacggcggcggggg
gtcgctgtacgggggcggcggttcgtccgggccgggccgggacggcgggggtccagccg
ggtcaggttgtccaccacggtggcggccgtggcggaggccagggcggcggtggtgtgtcc
ggcgcggcgggaacggcggaaggagaaccgctggaccggcggcatgtcgccggacacggc
```

## Promoter region *sco4253-54*

```
ttcagcgggccggtcggggcgagaccgacgaaggccgccaccgacgtgcccactccctcg
atcgggcgcgagccgctggccacctcctcgacgtagacgccgggcgacaggtaggacggc
atgctctgctctcctcggggtacgagacaggacgcccctcagcctcacgcgcgcgttgcc
gatgccgaaacgtccccgggtgccggagcgggggcatgttccttgcccctggtcgtgccc
gttggggcagactcccgccgcatcccggcacttcccccgaagacccctggcggacgccc
ggcgtttctgattcaacgtcttgtgtcgactgtcagttcgccagaatgtcatcttgcgat
caagccccgtttcggggcgccccgtcccgccgcaccggggcgctgccccgcgaagagggg
acggtgacggcgtgtcaggaggggaccgtccgtgtcggaccgcttgaaccagacgcacag
gcaccgcccggccggggccgctccggctccggcccgcccgcaggcacggcagtcgccgcc
gctggcctcgttgcaggccgcggcggggaaccgggccgtggcctcggcgatccagcgcgc
ggccggtaccgcacagctctccaaggccccggcaccggaacgggcgacgacctcgtcgac
gccggacgacctgcagatcacaccggtgacggagcccgagcagcaggcggccgtggacga
```

**Supplementary Fig. 7| *Sco* eCIS promoter regions.** The DNA sequence of the eCIS promoter regions of *Sco* is shown as diagrammed in Fig. 1b. The two divergent promoter regions are indicated by red blocks[1]. The translational start codons (for *sco4248* and *sco4249* on the top and *sco4253* and *sco4254* on the bottom) are underlined in red. Primers used to clone the promoter regions into the *luxCDABE* reporter plasmid are highlighted in yellow and indicated by arrows above the sequence.

## Supplementary Tables

| eCIS protein | WT | ΔBP | WT | ΔBP |
|---|---|---|---|---|
| | Intracellular | | Extracellular | |
| TR (DUF4255) | | | | |
| Tail sheath | | | | |
| Tail tube | | | | |
| BH1 | | | | |
| BH2 | | | | |
| BW1 | | | | |
| BW2 | | | | |
| BW3 | | | | |
| Sco4242 | | | | |
| Sco4251 | | | | |
| Sco4256 | | | | |
| ATPase | | | | |

**Supplementary Table 1| Summary of LC-MS/MS analysis of purified *Sco* eCIS samples.** eCIS-associated proteins identified through LC MS/MS analysis of purified *Sco* eCIS particles (Intracellular) or in concentrated purified extracellular media (Extracellular). Hits detected in three separate purifications are highlighted in dark grey. Hits identified in fewer than three experiments are highlighted in light grey. Only proteins with E-values better than $10^{-5}$ were considered a hit. Source data have been deposited in the MassIVE database under accession code; MSV000091288 [doi:10.25345/C5CC0V388].

| Gene name | Protein accession | Function | log(e) | log(I) | % Coverage | Total spectra | Molecular mass |
|---|---|---|---|---|---|---|---|
| Sco4260 | NP_733632 | Terminator / DUF4255 | -46.6 | 6.91 | 42 | 5 | 24.7 |
| Sco4253 | NP_628427.1 | Tail sheath | -251.7 | 8.22 | 77 | 77 | 56.9 |
| Sco4252 | NP_628426.1 | Tail tube | -150 | 7.57 | 83 | 40 | 16.5 |
| Sco4246 | NP_628420.1 | BH2/spike | -65 | 6.62 | 24 | 7 | 68 |
| Sco4244 | NP_628418.1 | BW2/gpJ | -190.1 | 7.52 | 52 | 25 | 70.8 |
| Sco4242 | NP_628416.1 | | -46.7 | 6.98 | 26 | 8 | 48.5 |
| Sco4256 | NP_628430.1 | Hydrolytic | -59.4 | 6.95 | 27 | 8 | 51,3 |

**Supplementary Table 2| Detailed results of LC-MS/MS analysis of WT eCIS samples.** Wild-type *Sco* were purified as described in Methods. The samples were reduced, trypsin digested and C18 purified before LC-MS and data processing. Values shown are from one representative run.
Log(e): the base-10 log of the expectation that any protein assignment was made at random.
Log(I): the base-10 log of the sum of the fragment ion intensities in the tandem mass spectra used to make this assignment.
% Coverage: the amino acid coverage of the protein in this assignment divided by the coverage corrected for peptide sequences that are unlikely to be observed using normal proteomics methods.
Total spectra: the total number of tandem mass spectra that can be assigned to this protein.

**Supplementary Table 3: Strains and plasmids used in this study.**

| **Strains** | **Description** | **Reference or source** |
|---|---|---|
| ***Escherichia coli* strains** | | |
| DH5α | | Stratagene |
| ET12567/pUZ8002 | Conjugation donor strain, $Kan^R$, $Cml^R$ | [2] |
| BW25113/pIJ790 | $\lambda$ Red recombination strain, $Cml^R$ | [3] |
| BL21(DE3) | Protein expression | Stratagene |
| Stellar HST08 | High transformation efficiency competent cells | Clontech |
| ***Streptomyces coelicolor* strains** | | |
| M145 | Wild-type *S. coelicolor* A3(2) | |
| WT::pf*lux*-EV | Promoterless *luxCDABE* operon | [4] |
| WT::pf*lux*-hrdBp | *luxCDABE* operon driven by the hrbB sigma factor promoter | [4] |
| WT::pf*lux*-redDp | *luxCDABE* operon driven by the RedD promoter | [4] |
| WT::pf*lux*-eCISp1 | *luxCDABE* operon driven by the eCIS promoter P1 | This study |
| WT::pf*lux*-eCISp2 | *luxCDABE* operon driven by the eCIS promoter P2 | This study |
| WT::pf*lux*-eCISp3 | *luxCDABE* operon driven by the eCIS promoter P3 | This study |

| **Strains** | **Description** | **Reference or source** |
|---|---|---|
| WT::pf*lux*-eCISp4 | *luxCDABE* operon driven by the eCIS promoter P4 | This study |
| M1152 | Derivative of M1146, Δ*act*, Δ*red*, Δ*cpk,* Δ*cda*, *rpoB* [C1298T] | [5] |
| M145 ΔTS (*Δsco4253::apra*) | eCIS tail sheath gene *sco4253* replaced with apramycin resistance cassette | This study |
| M145 ΔBP (Δ*sco4243*Δ*sco4244*Δ*sco4245::apra*) | eCIS baseplate genes *sco4243-45* replaced with apramycin resistance cassette | This study |
| M145 ΔATPase (Δ*sco4259::apra*) | eCIS ATPase gene *sco4259* replaced with apramycin resistance cassette | This study |
| M1152 ΔTS (Δ*sco4253::apra*) | | This study |
| M145::pIJ6902-actR-FLAG | M145 conjugated with pIJ6902 plasmid encoding inducible FLAG-tagged ActR (Cytoplasmic control) | [6] |
| M145::pIJ6902-S4646-FLAG | M145 conjugated with pIJ6902 plasmid encoding inducible FLAG-tagged SecE (Membrane control) | [6] |
| **Plasmids** | | |
| pFlux | Reporter plasmid, Promoterless luxCDABE operon, ApraR | [4] |

| **Strains** | **Description** | **Reference or source** |
|---|---|---|
| pFlux-eCISp1 | eCIS promoter region P1 (Sco4248-4249 reverse) | This study |
| pFlux-eCISp2 | eCIS promoter region P2 (Sco4248-4249 forward) | This study |
| pFlux-eCISp3 | eCIS promoter region P3 (Sco42453-4254 reverse) | This study |
| pFlux-eCISp4 | eCIS promoter region P4 (Sco42453-4254 forward) | This study |
| pIJ7331 | pBluescript KS (+), aac(3)IV, oriT (RK2) | [7] |
| p15TV-L | T7 expression vector, derivative of pET15b, AmpR, (GenBank ID: EF456736.1) | Novagen |
| p15-SC4245 | 6XHis BW1 expression plasmid | This study |
| p15-SC4253 | 6XHis tail sheath expression plasmid | This study |
| Cosmids | | |
| StD8a | | [8] |
| StD8aΔsco4253::apra | | This study |
| StD8aΔsco4243-45::apra | | This study |
| StD8aΔsco4259::apra | | This study |

**Supplementary Table 4: Oligonucleotides used in this study.**

| Oligonucleotide | Sequence (5'– 3') |
|---|---|
| **Lux reporter cloning** | |
| SCO_eCISp1p2GEN_F | GCTGCCAGACGAAGCCGTTGTTGCCGC |
| SCO_eCISp1p2GEN_R | CACCGTGGTGGACAACCTGACCCGGCT |
| SCO_eCISp1p2EcoRV_F | GGGATATCGGATCCCAGACGAAGCCGTTGTTGCCGC |
| SCO_eCISp1p2EcoRV_R | CCGATATCTGGTGGACAACCTGACCCGGCT |
| SCO_eCISp3p4GEN_F | GCGAGACCGACGAAGGCCGCCACCGA |
| SCO_eCISp3p4GEN_R | GGGCCTTGGAGAGCTGTGCGGTACCGGC |
| SCO_eCISp3p4EcoRV_F | GGGATATCGGATCCGACCGACGAAGGCCGCCACCGA |
| SCO_eCISp3p4EcoRV_R | CCGATATCTTGGAGAGCTGTGCGGTACCGGC |
| **Gene disruption** | |
| SCO4253_Disrp_F | GGGGCGTCCTGTCTCGTACCCCGAGGAGAGCAGAGCATG ATTCCGGGGATCCGTCGACC |
| SCO4253_Disrp_R | TAGAGGATGTGGGACAAGGGCGGGGGCGCGATCCGCCTA TGTAGGCTGGAGCTGCTTC |
| SCO_BP_Disrp_F | GACGAAGCGTTTCCGAGGAAGGCAGGTTGCTCCCTGATGA TTCCGGGGATCCGTCGACC |
| SCO_BP_Disrp_R | CCGCGGGGGTTCCGCACTCGGCGCAGTTCTGCGTGGTCAT GTAGGCTGGAGCTGCTTC |
| SCO4259_Disrp_F | CGTGACGGACGGCCGCACGAAACGACAGTGACGACCGTG ATTCCGGGGATCCGTCGACC |
| SCO4259_Disrp_R | AAGAAGGGCTGACGGCCGGAGGGGGCCCGGCGGCCGTCA TGTAGGCTGGAGCTGCTTC |

| **Oligonucleotide** | **Sequence (5'– 3')** |
|---|---|
| **Gene replacement verification** | |
| StD8aΔTS_aac(3)IV _F | GAAGTGCCGGGATGCG |
| StD8aΔTS_aac(3)IV _R | GCAGGTTGGAGACCGAGTTG |
| StD8aΔBP_aac(3)IV _F | CGTCAACGTGCAGATCAACTCC |
| StD8aΔBP_aac(3)IV _R | CGAAGGCGTCGAAGCCGGGGCG |
| StD8aΔATP_aac(3)IV _F | CGACCCGCCGATGTCCGAGG |
| StD8aΔATP_aac(3)IV _R | CGGCGCCCTCTTCTCCAAG |
| **Recombinant protein cloning** | |
| p15TV-L SCgpW_F | TTGTATTTCCAGGGCATGGCCGAACAGTTCGTC |
| p15TV-L SCgpW_R | CAAGCTTCGTCATCATCAGCGGTCGCTTTCG |
| p15TV-L SC_Vdmn_F | TTGTATTTCCAGGGCATGGTCGCCAACGCCATC |
| p15TV-L SC_Vdmn_R | CAAGCTTCGTCATCA GTCGCTCTTGGTCGGC |
| p15TV-L SCTS_F | TTGTATTTCCAGGGCATGCCGTCCTACCTGTCG |
| p15TV-L SCTS_R | CAAGCTTCGTCATCACTACTCGTCCAGTTCGCCG |

## Supplementary References


1. Kim, D. W., Chater, K. F., Lee, K. J. & Hesketh, A. Effects of growth phase and the developmentally significant bldA-specified tRNA on the membrane-associated proteome of Streptomyces coelicolor. *Microbiology (Reading)* **151**, 2707-2720, doi:10.1099/mic.0.28000-0 (2005).
2. Flett, F., Mersinias, V. & Smith, C. P. High efficiency intergeneric conjugal transfer of plasmid DNA from Escherichia coli to methyl DNA-restricting streptomycetes. *FEMS Microbiol Lett* **155**, 223-229, doi:10.1111/j.1574-6968.1997.tb13882.x (1997).
3. Datsenko, K. A. & Wanner, B. L. One-step inactivation of chromosomal genes in Escherichia coli K-12 using PCR products. *Proc Natl Acad Sci U S A* **97**, 6640-6645, doi:10.1073/pnas.120163297 (2000).
4. Craney, A. *et al.* A synthetic luxCDABE gene cluster optimized for expression in high-GC bacteria. *Nucleic Acids Res* **35**, e46, doi:10.1093/nar/gkm086 (2007).
5. Gomez-Escribano, J. P. & Bibb, M. J. Engineering Streptomyces coelicolor for heterologous expression of secondary metabolite gene clusters. *Microb Biotechnol* **4**, 207-215, doi:10.1111/j.1751-7915.2010.00219.x (2011).
6. Craney, A., Tahlan, K., Andrews, D. & Nodwell, J. Bacterial transmembrane proteins that lack N-terminal signal sequences. *PLoS One* **6**, e19421, doi:10.1371/journal.pone.0019421 (2011).
7. Gust, B., Kieser, T., and Chater, K.F. REDIRECT Technology. PCR Targeting Systems in Streptomyces coelicolor (2002).
8. Redenbach, M. *et al.* A set of ordered cosmids and a detailed genetic and physical map for the 8 Mb Streptomyces coelicolor A3(2) chromosome. *Mol Microbiol* **21**, 77-96, doi:10.1046/j.1365-2958.1996.6191336.x (1996).
