# Supplementary material for: A contractile injection system is required for developmentally regulated cell death in Streptomyces coelicolor
Source: Nat Commun. 2023 Mar 16;14:1469. doi: 10.1038/s41467-023-37087-7 (PMC10020575; doi:10.1038/s41467-023-37087-7)
Supplement: Supplementary file 2 — Description of Additional Supplementary Files [file 41467_2023_37087_MOESM2_ESM.pdf]

## Description of Additional Supplementary Files:

**Supplementary Data 1|** Summary of bioinformatically predicted elements in subtype IId Streptomyces eCIS. Table summarizing the presence of newly predicted features conserved across 105 type IId eCIS in Streptomyces that were analyzed in this study. Fiber identification and analysis is detailed in Supplementary Data 6 and 7. Effector identification and analysis is detailed in Supplementary Data 4 and 5. Genes containing TTA codons within eCIS clusters are provided in Supplementary Data 2.

**Supplementary Data 2|** List of all genes containing a leucine encoding TTA within eCIS of Streptomyces. Nucleotide sequences of Streptomyces eCIS, including 10 kb regions flanking the cluster in both directions, were manually analyzed for the presence of in-frame TTA codons, encoding for leucine residues within annotated ORFs. The function of the TTA16 containing gene was determined using protein annotation on NCBI and HMM-based annotations using the PAT server.

**Supplementary Data 3|** eCIS genomic loci in the Streptomyces genus. The genomic eCIS loci of 127 Streptomyces genomes are shown. Colored blocks represent ORFs annotated to have a conserved eCIS function, detailed in the legend. White blocks are ORFs of unknown or unrelated function. eCIS subtype (Ia-IId) has been assigned based on the classification of Chen et al. (2019) and is indicated in the second column. MPTase is a metallopeptidase domain that is often associated with eCIS toxins.

**Supplementary Data 4|** BLAST search results for homologues of Sco4256, the putative hydrolytic effector protein in subtype IId eCIS. Values were obtained by BlastP (Sayers et al., 2021) with the sequence of AKZ56493.1 from Streptomyces ambofaciens ATCC 23877 used as query. AKZ56493.1 is closely related to Sco4256 (76% identical). The presence of a predicted pore-forming lectin domain for each protein sequence was determined by HHpred (Soding et al., 2005) and is indicated by (+).

**Supplementary Data 5|** HHpred Graphical Output for Sco4256 search. An alignment of diverse homologs of Sco4256 was used as query and analyzed for structural similarity prediction by HHpred. The output graphical representation provided by the HHpred server of the top hits for this search is shown. The three predicted domains are indicated above the image. The best predicted hits for each domain, including probability and P-values, are shown below.

**Supplementary Data 6|** BLAST search results for homologues of the putative fiber in Streptomyces eCIS. Values were obtained by BlastP with the putative fiber sequence of Streptomyces albus J1074 (YP_007743954.1) used as query. YP_007743954.1 is closely related to the Sco putative fiber (72% identical).

**Supplementary Data 7|** HHpred Graphical Output for Sco4242 search. An alignment of diverse homologs of Sco4242 was used as query and analyzed for structural similarity prediction by HHpred. The output graphical representation provided by the HHpred server of the top hits for this search is shown. The three predicted domains are indicated above the image. The best predicted hits for each domain, including probability and P-values, are shown below.
